# Genomics and transcriptomics of epizoic Seisonidea (Rotifera, syn. Syndermata) reveal strain formation and gradual gene loss with growing ties to the host

**DOI:** 10.1186/s12864-021-07857-y

**Published:** 2021-08-09

**Authors:** Katharina M. Mauer, Hanno Schmidt, Marco Dittrich, Andreas C. Fröbius, Sören Lukas Hellmann, Hans Zischler, Thomas Hankeln, Holger Herlyn

**Affiliations:** 1 Institute of Organismic and Molecular Evolution (iomE), Anthropology, Johannes Gutenberg University Mainz, Mainz, Germany; 2 Molecular Andrology, Biomedical Research Center Seltersberg (BFS), Justus Liebig University Gießen, Giessen, Germany; 3 Institute of Organismic and Molecular Evolution (iomE), Molecular Genetics and Genomic Analysis Group, Johannes Gutenberg University Mainz, Mainz, Germany

**Keywords:** Genome, Transcriptome, Rotifera, Syndermata, Seisonacea, Seisonidae, Mitogenome, Cryptic speciation, Evolution of parasitism, Long branch attraction

## Abstract

**Background:**

Seisonidea (also Seisonacea or Seisonidae) is a group of small animals living on marine crustaceans (*Nebalia* spec.) with only four species described so far. Its monophyletic origin with mostly free-living wheel animals (Monogononta, Bdelloidea) and endoparasitic thorny-headed worms (Acanthocephala) is widely accepted. However, the phylogenetic relationships inside the Rotifera-Acanthocephala clade (Rotifera *sensu* *lato* or Syndermata) are subject to ongoing debate, with consequences for our understanding of how genomes and lifestyles might have evolved. To gain new insights, we analyzed first drafts of the genome and transcriptome of the key taxon Seisonidea.

**Results:**

Analyses of gDNA-Seq and mRNA-Seq data uncovered two genetically distinct lineages in *Seison nebaliae* Grube, 1861 off the French Channel coast. Their mitochondrial haplotypes shared only 82% sequence identity despite identical gene order. In the nuclear genome, distinct linages were reflected in different gene compactness, GC content and codon usage. The haploid nuclear genome spans ca. 46 Mb, of which 96% were reconstructed. According to ~ 23,000 SuperTranscripts, gene number in *S. nebaliae* should be within the range published for other members of Rotifera-Acanthocephala. Consistent with this, numbers of metazoan core orthologues and ANTP-type transcriptional regulatory genes in the *S. nebaliae* genome assembly were between the corresponding numbers in the other assemblies analyzed. We additionally provide evidence that a basal branching of Seisonidea within Rotifera-Acanthocephala could reflect attraction to the outgroup. Accordingly, rooting via a reconstructed ancestral sequence led to monophyletic Pararotatoria (Seisonidea+Acanthocephala) within Hemirotifera (Bdelloidea+Pararotatoria).

**Conclusion:**

Matching genome/transcriptome metrics with the above phylogenetic hypothesis suggests that a haploid nuclear genome of about 50 Mb represents the plesiomorphic state for Rotifera-Acanthocephala. Smaller genome size in *S. nebaliae* probably results from subsequent reduction. In contrast, genome size should have increased independently in monogononts as well as bdelloid and acanthocephalan stem lines. The present data additionally indicate a decrease in gene repertoire from free-living to epizoic and endoparasitic lifestyles. Potentially, this reflects corresponding steps from the root of Rotifera-Acanthocephala via the last common ancestors of Hemirotifera and Pararotatoria to the one of Acanthocephala. Lastly, rooting via a reconstructed ancestral sequence may prove useful in phylogenetic analyses of other deep splits.

**Supplementary Information:**

The online version contains supplementary material available at 10.1186/s12864-021-07857-y.

## Background

Seisonidea (also Seisonacea or Seisonidae) includes worm-like creatures up to 2.5 mm in size that live in marine environment on opossum shrimps (*Nebalia* spec., Crustacea, Leptostraca) [1–4]. Traditionally, Seisonidea is subsumed under Rotifera or wheel animals, along with Monogononta and Bdelloidea the species of which are mostly free-living [5]. However, as already noticed by Grube in 1861 [1], there are considerable differences between seisonids and other wheel animals regarding their morphology. Perhaps the most striking one is that seisonids have small ciliary brushes on each side of the mouth opening, but lack a rotatory or wheel organ (also corona), which in monogononts and bdelloids enables swimming and swirling food to the mouth [1–3, 6]. Seisonids are further distinguished by the continuous occurrence of well-developed males in addition to females and strictly sexual reproduction [1, 2, 6]. In contrast, males are absent in bdelloids and the sporadically occurring males in monogononts are dwarfed and morphologically simplified [3, 5, 7]. Nevertheless, Monogononta and Bdelloidea clearly belong to the closer phylogenetic relatives of Seisonidea – and they are joined by thorny-headed worms (Acanthocephala), i.e., endoparasites of mandibulate arthropods and gnathostome vertebrates [8, 9]. A monophyletic origin of these four taxa was probably proposed earliest in the middle of the previous century [10] and gained further impetus from ultrastructural studies in the 1990s [11–13]. The members of the group share a syncytial organization of the tegument (epidermis). Referring to this evolutionary novelty, the taxon is designated as Syndermata in part of the scientific literature (e.g., [14, 15]), while other authors prefer to subsume Acanthocephala under broader Rotifera (e.g., [16]). The monophyly of the group, which we here follow others in calling Rotifera-Acanthocephala [17], is in any case confirmed by numerous molecular analyses (e.g., [14, 16, 18, 19]).

With the growing number of genome assemblies, it has become increasingly clear that genome and transcriptome metrics vary considerably within Rotifera-Acanthocephala. Examples include haploid genome size estimates of 55 or 211 Mb in monogononts and of 122 Mb or larger in bdelloids [20–22]. The latter value is close to approximately 260 Mb of the first acanthocephalan draft genome published [23]. However, while bdelloid genomes carry signatures of genome duplication (e.g., [20]), the organization of the acanthocephalan nuclear genome primarily testifies to expansion of the repetitive portion [23]. Similarly, the proliferation of transposable elements was considered to be causative for the larger genomes inside Monogononta [22]. This complexity impairs deriving a scenario of genome evolution within Rotifera-Acanthocephala. Further hampering the issue is the lack of comprehensive comparative data on a key taxon, the Seisonidea. In fact, for seisonids, solely mitochondrial gene sequences [14, 16, 18], a set of expressed sequence tags [19] and one mitochondrial genome [24] were available previous to this study. Reconstruction of first seisonid genome and transcriptome drafts would therefore significantly broaden the data basis for evolutionary analyses of Rotifera-Acanthocephala.

A broader molecular data basis might also shed new light on diversity within seisonids, for which four species have been described so far [1, 2, 25, 26]. In comparison, numbers of described species are many times higher for Monogononta (1570 species [27]), Bdelloidea (461 [27]) and Acanthocephala (ca. 1200 [28]). And these counts are growing, which partially reflects the discovery or resurrection of morphologically distinguished species (e.g., [29, 30]). For example, there is increasing evidence that the acanthocephalan *Pomphorhynchus laevis* might be a collective species containing several morphologically distinct lineages [23, 31]. In addition, molecular analyses have uncovered more and more cryptic species in monogononts, bdelloids and acanthocephalans [32–34]. Only for seisonids, cryptic speciation has not yet been reported although it might occur in this group as well. Grube, for example, distinguished four seisonid species in the Gulf of Naples, Italy, alone [35]. And while this view has not gained acceptance, it is clear that different seisonid species can coexist on single opossum shrimps. This illustrates that co-existence of genetically distinct lineages is possible in seisonids [2].

Comprehensive genomic data may further elucidate the phylogenetic position of seisonids within the Rotifera-Acanthocephala clade. In fact, seisonids appeared as the acanthocephalan sister-group in part of the sequence-based analyses, especially when implementing measures for mitigating potential long branch attraction (LBA) [18, 23]. Such position of seisonids would be in accordance with mitochondrial gene order and ultrastructural data [11, 12, 24, 36] and also with combined analysis of morphological and molecular data [37]. A monophylum containing Seisonidea and Acanthocephala may be referred to as Pararotatoria Sudzuki, 1964, a taxon name originally applied to Seisonacea or Seisonidea alone [5]. Pararotatoria, in turn, could be the sister-group of Bdelloidea, which together would establish Hemirotifera (Bdelloidea+Pararotatoria) [19, 24, 37]. Yet, seisonids were basally branching in other molecular analyses, thus being sister to a clade comprised of monogononts, bdelloids and acanthocephalans [14, 16, 38]. In the respective trees, bdelloids grouped together with acanthocephalans, implying monophyly of Lemniscea as suggested by Lorenzen [39]. Correspondingly, the phylogeny inside the Rotifera-Acanthocephala clade has repeatedly been regarded as unsettled [4, 5]. Moving further to unravel the phylogenetic relationships of monogononts, bdelloids, seisonids and acanthocephalans would thus be welcome and all the more so, as it should lead to a better understanding of the evolution of genomes and lifestyles inside the entire clade.

The present study aims at shedding light on the aforementioned issues. For achieving this, we present the first nuclear genome and transcriptome drafts for Seisonidea, as generated from next generation sequencing (NGS) data. We additionally screened for potential hidden diversity within specimens morphologically determined as *S. nebaliae* Grube, 1861 [1]. Furthermore, we compared genome and transcriptome metrics within Rotifera-Acanthocephala. Finally, we re-evaluate the phylogenetic position of Seisonidea under particular consideration of potential LBA. Based on the results of tree reconstructions, we infer scenarios of how genomes and lifestyles might have evolved inside the Rotifera-Acanthocephala clade.

## Results

### Two mitochondrial genomes in pooled *S. nebaliae* sample

**Fig. 1:**
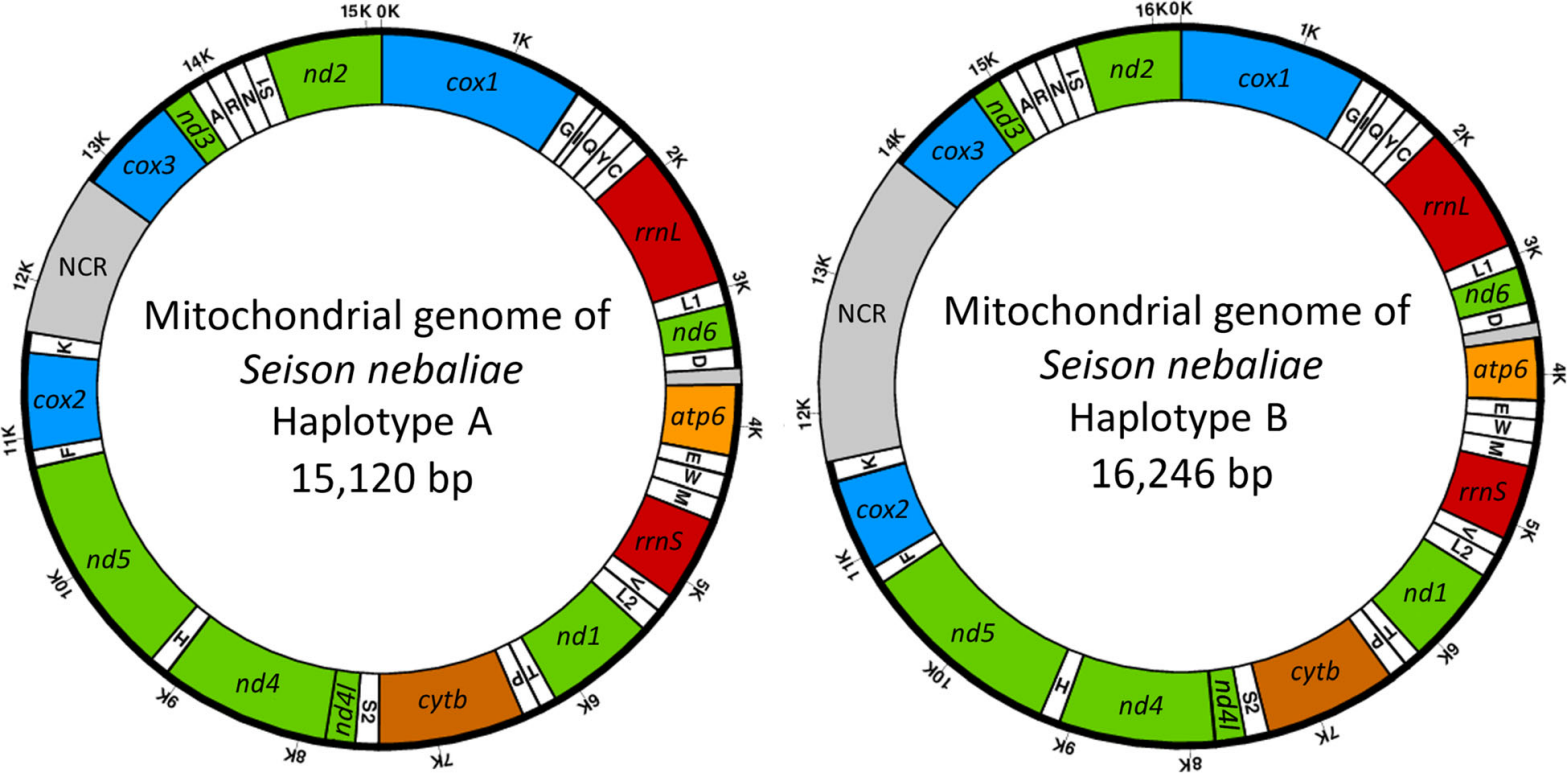
Schematic depiction of mitochondrial haplotypes A and B (right) in *S. nebaliae*. Both mitochondrial sequences contain 12 protein-coding genes in the same order on the heavy strand. These are in alphabetical order (encoded proteins behind commas): *atp6*, ATP synthase subunit 6; *cox1–3*, cytochrome c oxidase subunits 1–3; *cytb*, cytochrome b; *nd1–6*, NADH dehydrogenase subunits 1–6; *nd4l*; NADH dehydrogenase subunit 4l. Genes *rrnS* and *rrnL* code for 12S and 16S rRNAs, respectively. White highlights tRNA genes for the twenty canonical amino acids as given in one-letter code (e.g. trnA). tRNA genes for serine (S) and lysine (L) have two copies, each. Different lengths of both mitogenomes are due to a longer non-coding regions (NCR) in haplotype B

**Fig. 2:**
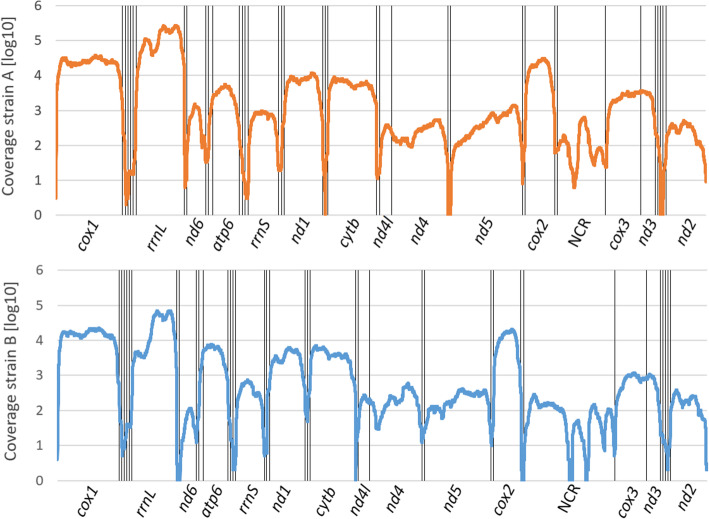
RNA coverage (multiplicity) of *S. nebaliae* haplotypes A and B (below). Positional read depth is increased in protein-coding and rRNA genes in both haplotypes. Positions without mapped reads were assigned a value of 0. Vertical lines correspond to gene boundaries. *atp6*, ATP synthase subunit 6; *cox1–3*, cytochrome c oxidase subunits 1–3; *cytb*, cytochrome b; *nd1–6*, NADH dehydrogenase subunits 1–6; *nd4l*; NADH dehydrogenase subunit 4l; *rrnS*, 12S rRNA and *rrnL,* 16S rRNAs

MEGAHIT assembled two mitochondrial sequences based on DNA from 594 specimens determined morphologically to belong to *S. nebaliae*. With 15,114 bp, one of the two sequences was almost identical in length to the 15,120 bp long mitochondrial genome previously published for *S. nebaliae* (KP742964.1). As identity was high between these two sequences (99.9%), we collectively denote these as haplotype A and refer to Sielaff et al. [24] for a detailed description. According to the best hit from BLASTN (for references to programs see Methods), the second mitogenome sequence belonged to *S. nebaliae* too. However, with ca. 82.0% this consensus sequence showed considerably less nucleotide identity with the previously published reference (based on an alignment of 14,993 bp as compiled with MAFFT and Gblocks). In the following, we regard the corresponding mitogenome as haplotype B. Both haplotypes contained the 12 protein-coding genes, 2 rRNA genes and 22 tRNA genes to be expected for Gnathifera. Thus, tRNAs for lysine and serine were represented by two genes, each, while *atp8* was lacking (e.g., [40, 41]). Haplotypes A and B also shared an identical gene order and all genes were encoded by the heavy strand in both of them (Fig. 1). Furthermore, RNA-Seq reads (enriched for coding sequences) mapped to all rRNA- and protein-coding genes in both mitogenomes (Fig. 2), thus indicating conserved transcription. On the other hand, with 16,246 bp, haplotype B exceeded haplotype A by about 1130 bp, due to length polymorphism of the non-coding regions (NCRs). Across the entire sequence of haplotype B, average gDNA read depth was 9111 (Bowtie 2). With 648, read depth was approximately 14-fold lower in haplotype A. In turn, RNA reads mapped with lower multiplicity to haplotype B (average 4598) than haplotype A (average 14,012).

**Fig. 3:**
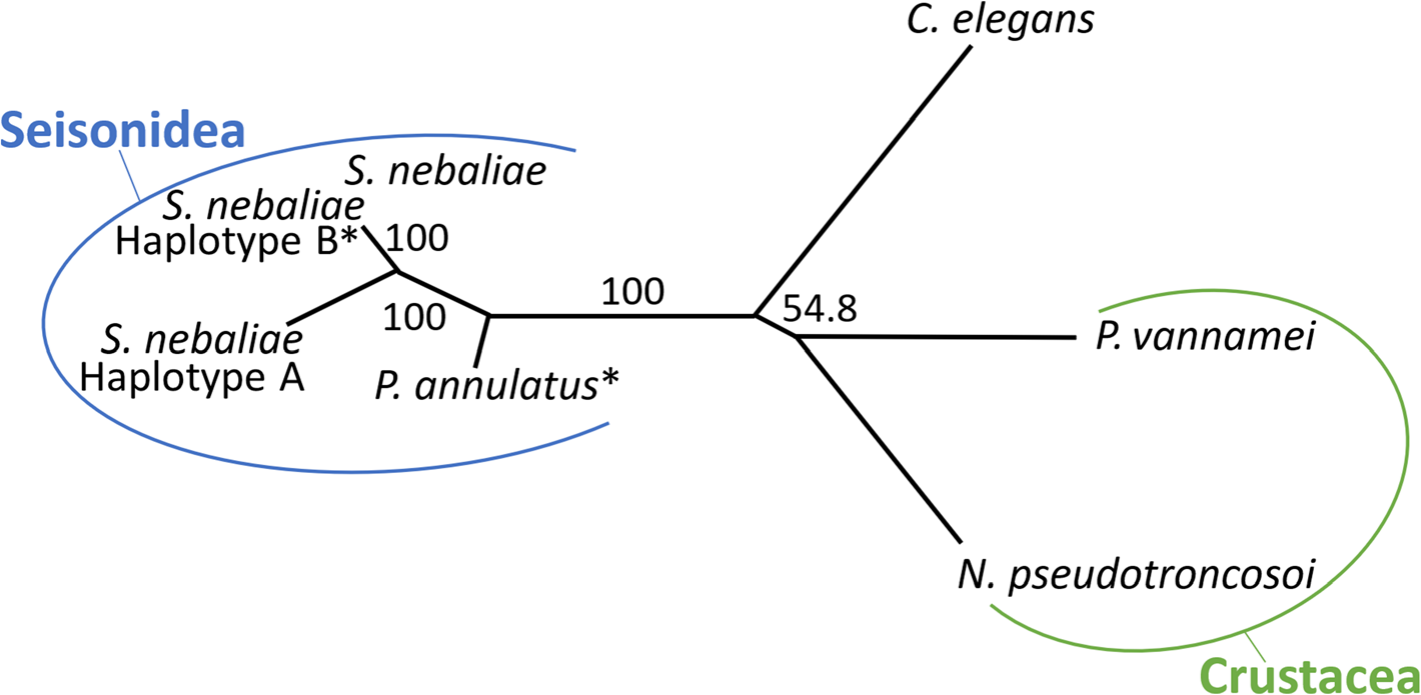
Unrooted Bayesian inference (BI) tree as reconstructed from a *cox1* alignment (643 bp). Sequences of *S. nebaliae* form a cluster, which again groups together with the *P. annulatus* sequence. In addition, the distance from the crustacean cluster (*Nebalia pseudotroncosoi* and *Penaeus vannamei*) to the branching of the nematode *Caenorhabditis elegans* is smaller than to the nodes, from which seisonid species branch off. Divergence of a previously published *cox1* sequence for *S. nebaliae* [37] and its newly discovered counterpart (haplotype B) is not resolved in the graphic due to high identity. Support values correspond to the frequency of a branch in the posterior distribution. Asterisks (*) highlight newly generated sequences. Tree reconstruction was conducted with MrBayes v. 3.2.7a based on the GTR-G substitution model. Visualization was performed using iTOL. For *nd1* tree, see Figure S1 in Additional file 1

Subsequently, we verified that the new haplotype B is attributable to *S. nebaliae* by phylogenetic analyses. Due to limited representation of orthologues in our WGA-based NGS dataset for *Paraseison annulatus *Claus, 1876 the approach was confined to *cox1* and *nd1* sequences. In unrooted maximum likelihood (ML) and Bayesian inference (BI) trees, none of the sequences from this study clustered with orthologues of crustaceans, thereunder a *Nebalia* species (see Supplementary Table S1, Additional file 1). In particular, all *Seison* sequences persistently formed a cluster, as exemplified by the BI tree in Fig. 3. Thus, the new mitogenome does not result from contamination of the sequenced sample with DNA of its host, *Nebalia bipes*, or the second seisonid species living on that crustacean, *P. annulatus*. Mitogenome-based tree reconstruction hence suggests variation within *S. nebaliae*. However, the extent of variation is so strong, given the 82% sequence identity mentioned above, that we consider the haplotypes found here as a first indication of distinct lineages within *S. nebaliae*.

### Metrics of genome assemblies provide further evidence for distinct lineages in *S. nebaliae*

The appearance of two different mitochondrial genomes led us to take into consideration that two distinct lineages (A and B) of *S. nebaliae* could be included in the data*.* To further examine this working hypothesis we applied two alternative strategies for assembling the nuclear genome with MEGAHIT. Nuclear genome assembly 1 (GA1) was reconstructed without stringent filtering against less abundant reads. The assembly should thus represent nuclear genomes A and B, besides potential foreign DNA. In contrast, genome assembly 2 (GA2) was intended to be built from genome B reads only. For achieving this, we applied a multiplicity threshold of 20. The threshold was a slight enhancement compared to the multiplicity ratio of mitochondrial haplotypes as derived by mapping of gDNA reads. 

Genome assembly 2 was more coherent compared to GA1 (Table 1). Specifically, GA2 had almost twice the N50 value of GA1 (ca. 45.7 kb in GA2, ca. 26.0 kb in GA1). Furthermore, GA2 contained disproportionally less contigs (3301 in GA2 versus 14,609 in GA1). Complementary search for BUSCO genes underlined better quality of GA2 relative to GA1 in fewer contigs of bacterial origin in the first relative to the latter (Table 1). This was additionally reflected in a lower error rate for GA2 relative to GA1, while the inverse relation held for the consensus quality value (Table 2). Nevertheless, Merqury gave the accuracy in consensus base calling with 99.9% (Q30) for GA1 and 99.99% (Q40) for GA2 (Table 2). Thus, the sequence information per se was of high quality in both assemblies.

**Table 1:** Characteristics of the *S. nebaliae* genome

Metric	GA1 Genomes A + B	GA2 Genome B
Number of contigs (minimum 1000 bp)	14,609	3301
Length [bp]	98,982,171	43,884,832
N50 [bp]	25,666	45,684
GC content [%]	34.0	31.9
BUSCO – Bacteria genes* [%]	M = 0.0, D = 82.3	M = 71.8, D = 1.6
BUSCO – Metazoa genes* [%]	M = 35.4, D = 26.9	M = 36.5, D = 0.3
Back-mapping rate of gDNA reads [%]	88.2	87.5
Mapping rate of mRNA reads [%]	91.8	32.8

*GA1* nuclear genome assembly 1, *GA2* nuclear genome assembly 2, *D* duplicated BUSCO genes, *M* missing BUSCO genes. Asterisks highlight references to BUSCO v. 4.0.6, database odb10

**Table 2:** Merqury metrics for drafts of the nuclear genome in *S. nebaliae*

Metric	GA1 Genomes A + B	GA2 Genome B
consensus quality value	31.825	46.780
Assembly error rate	6.569e-04	2.099e-05
k-mer completeness (%)	92.569	91.638

*GA1* nuclear genome assembly 1, *GA2* nuclear genome assembly 2

**Fig. 4:**
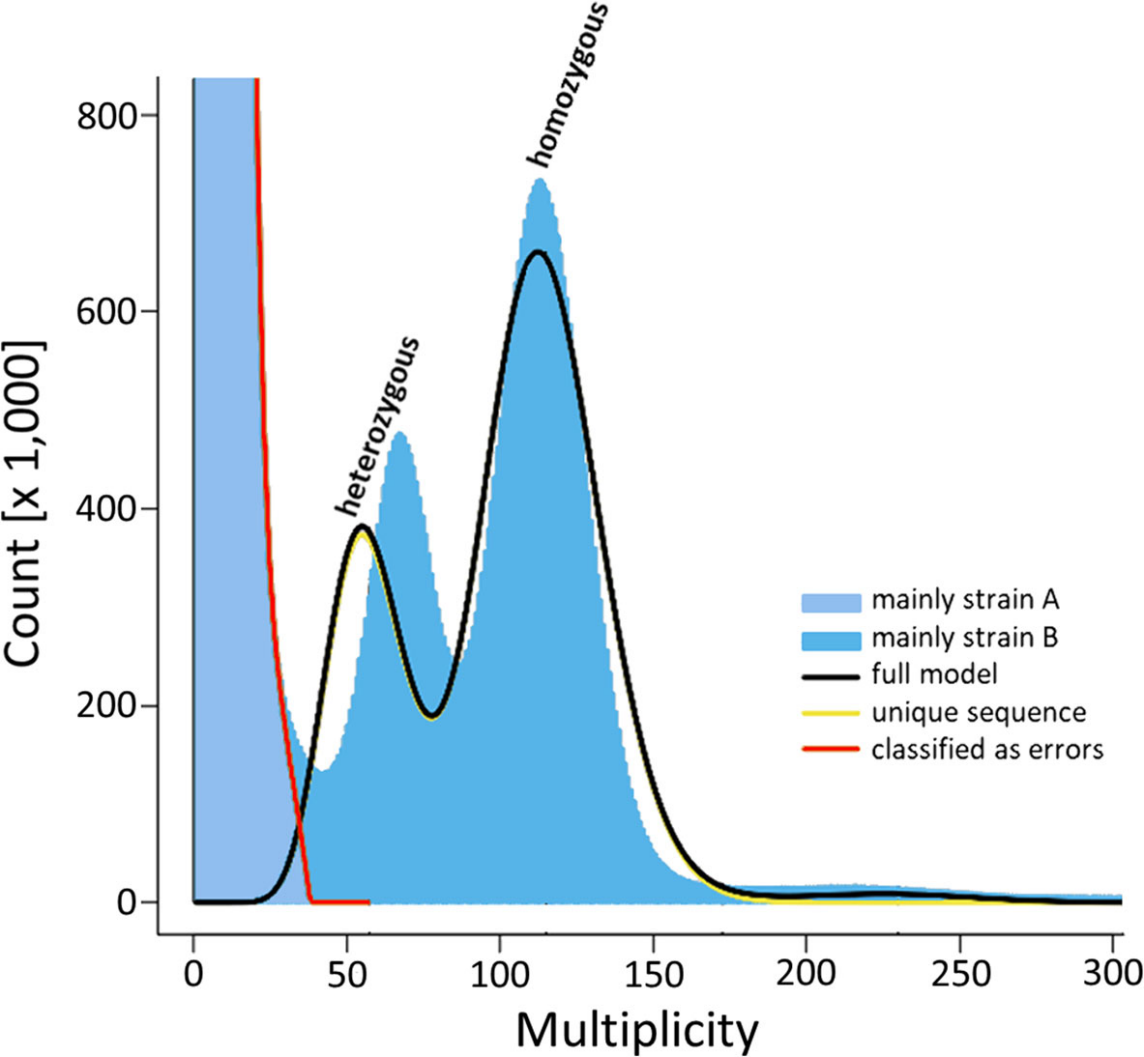
Frequency distribution of k-mers (k = 21 bp) in NGS reads from pooled *S. nebaliae* DNAs. According to GenomeScope, distributions were 73.6% identical between the processed NGS data and the diploid genome model. Observed k-mers left of the red line (light blue fill) were classified as errors due to their low frequencies but will also include k-mers from less abundant carriers of nuclear genome A. Blue fill right of the red line should correspond to k-mers derived of *S. nebaliae* specimens carrying nuclear genome B. The shift in multiplicity between observation (77 x) and the heterozygous peak in the diploid model (60 x) is likely due to genetic variation in lineage B. GenomeScope v. 1.0 [42] was run with trimmed and filtered DNA reads

**Fig. 5:**
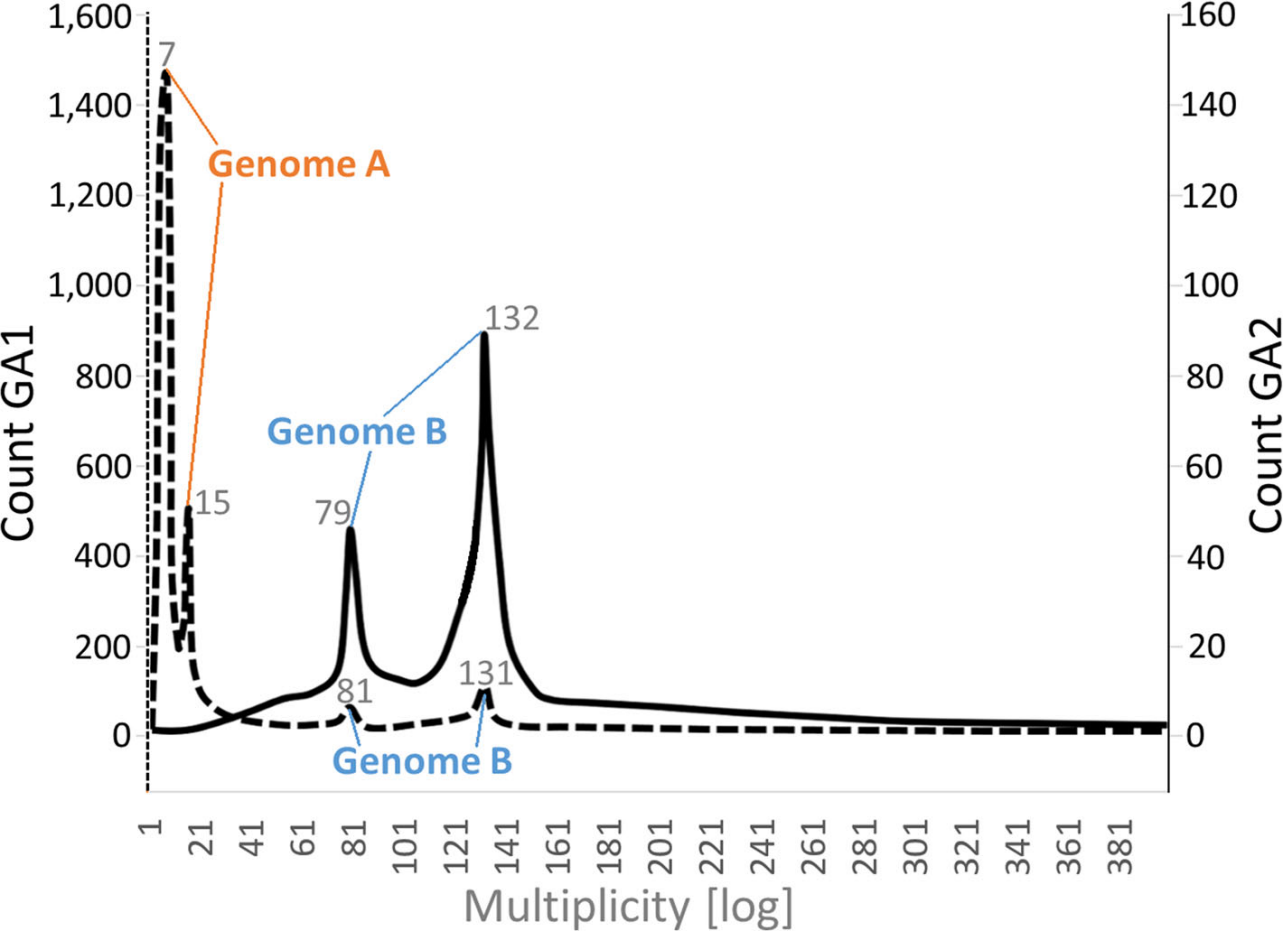
Approximate frequency of genomic contigs (*S. nebaliae*) in relation to multiplicity. The dashed line for nuclear genome assembly 1 (GA1) has two larger peaks at multiplicity values of 7 x and 15 x (Genome A) and two smaller ones at 79 x and 132 x (Genome B). After having filtered for high-abundance reads (≥ 20 x), two peaks at 81 x and 131 x (Genome B) remain in the solid line giving genome assembly 2 (GA2). Contigs with multiplicity values larger 401 x had negligible frequencies and hence are not shown. Multiplicity was averaged across contigs upon back-mapping of gDNA reads with Bowtie 2

**Fig. 6:**
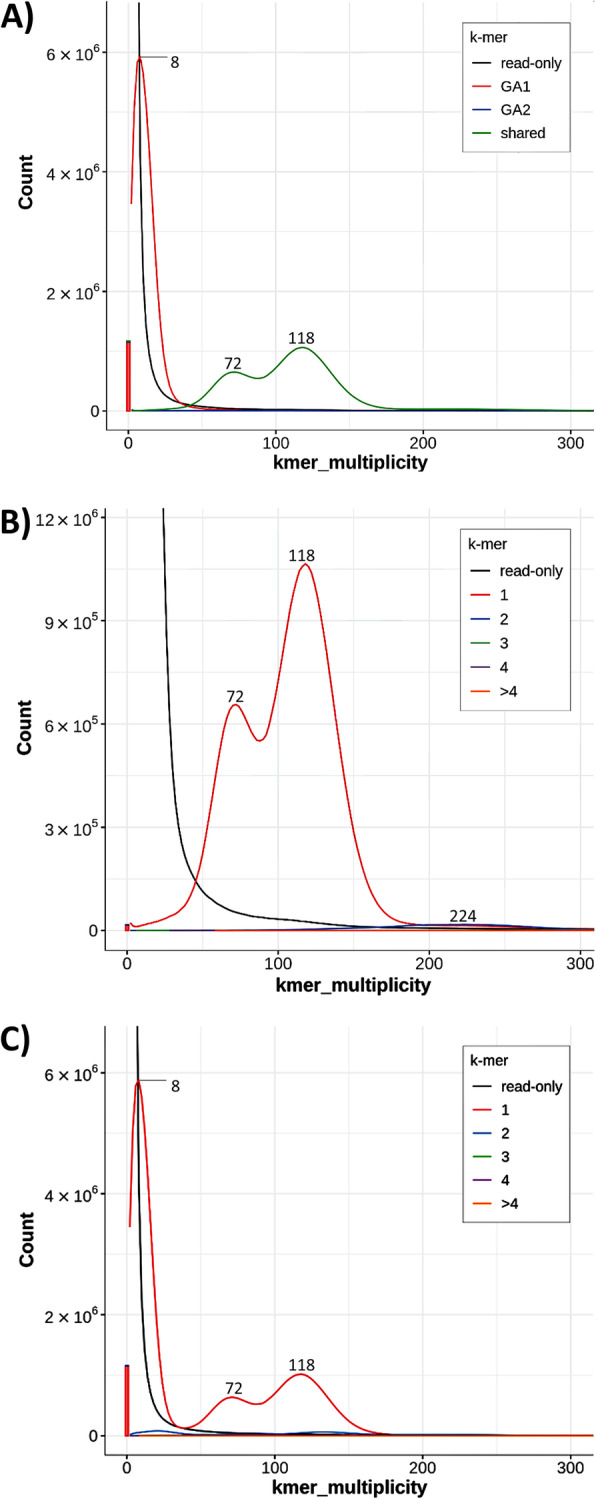
Frequency distributions of k-mers as inferred for assemblies of nuclear genomes in *S. nebaliae*. **A)** Combined spectra-asm plot for genome assemblies 1 (GA1) and 2 (GA2). The distribution for shared k-mers (green line) peaks at multiplicity values of 72 x and 118 x. The peak at a multiplicity of 8 x gives private k-mers derived from the nuclear genome A of *S. nebaliae* (red line). In contrast, private k-mers are absent in GA2 (blue curve along the ordinate). **B)** Spectra-cn plot for GA2. Peaks at multiplicity values of 72 x and 118 x should correspond to heterozygous and homozygous portions in haploid nuclear genome B. A smaller peak at multiplicity of 224 x represents k-mers occurring in duplicates. **C)** Spectra-cn plot for GA1. The frequency distribution (red line) combines the peak of private GA1 k-mers (genome A) and the peaks corresponding to heterozygous and homozygous k-mers in GA2 (genome B). Numbers in inserts of plots B and C refer to ploidy states. In each plot, the black line gives k-mers lacking matches in the contigs of GA1 and/or GA2. The bar at zero multiplicity gives the fraction of k-mers which is likely due to errors in consensus base calling, each. Colours in each bar refer to the code given in the inserts. As can be seen, the error rate is smallest in GA2. Analyses were carried out with Merqury

With about 43.9 Mb, the length of GA2 undershot the GenomeScope estimate by only 1.8 Mb. Correspondingly, GA2 should cover approximately 96% of the haploid nuclear genome (B) of *S. nebaliae*. Moreover, GA2 spanned less than half of the 99 Mb in GA1 (Table 1). This is consistent with the expectation for GA2 representing genome B mainly and GA1 including genomes A and B in addition to a smaller fraction of contigs assembled from foreign DNA. Near-to completeness of GA1 and GA2 was underlined by additional Merqury estimates. In particular, k-mer completeness was given with about 93% for GA1 and about 92% for GA2 (Table 2). The difference between both estimates was in line with the fact that k-mers originating from foreign DNA were less contained in GA2 than GA1. These were included in k-mer distributions in low-multiplicity areas (Figs. 4, 5 and 6A, C). Thus, the application of a multiplicity threshold (20 x) effectively tailored GA2 to contain genome B contigs.

**Fig. 7:**
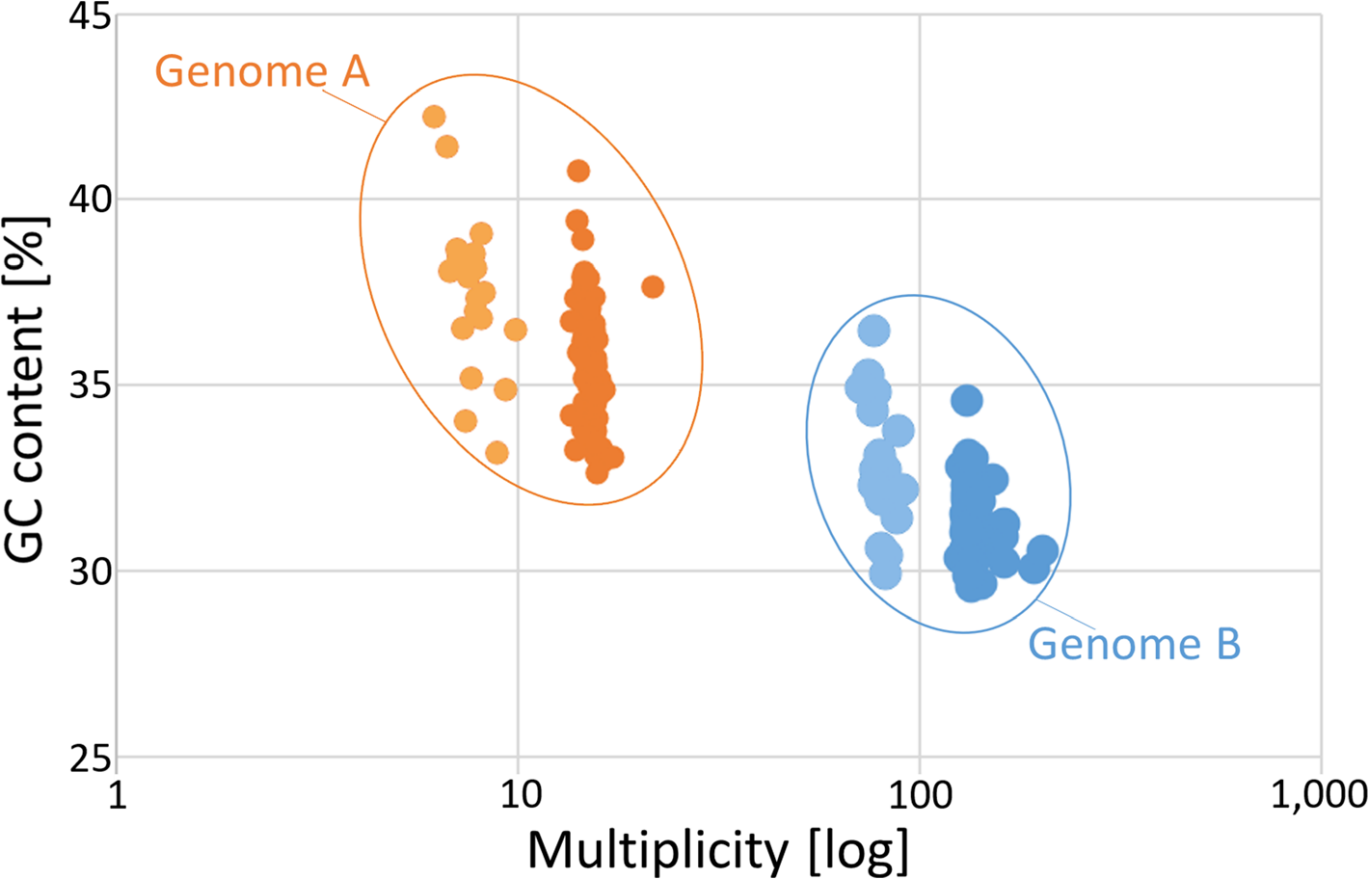
GC content of genomic contigs representing *S. nebaliae* genomes A and B, in relation to multiplicity. The level of GC content differs significantly between contigs giving genome A (*N* = 93) and their counterparts from genome B (*N* = 94) as revealed by MWU test (FDR = 0.000). Contigs classified as heterozygous (light fill) have slightly increased GC content compared to contigs classified as homozygous according to multiplicity. The contigs analyzed included 100 orthologous BUSCO Metazoa genes per lineage or strain. As some of the contigs contained multiple genes, 187 (= 93 + 94) entered analysis

Distinct lineages in *S. nebaliae* were further reflected in a higher GC content of GA1 (34.0%) than GA2 (31.9%) (Fig. 7) and the rates at which mRNA-Seq reads mapped to both genome assemblies. In fact, also the RNA used for NGS was extracted from a pool of *S. nebaliae* specimens and hence should represent both lineages, although not necessarily at the same ratio as the DNA pool. In line with this, the mapping rate in the case of GA2 (32.8%) was clearly smaller than in GA1 (91.8%). In contrast, back-mapping rates of gDNA reads prior and after application of a multiplicity cutoff (Bowtie 2) were very similar in both genome assemblies (Table 1).

Representation of two nuclear genomes in GA1 additionally emerged from a high proportion of duplicated BUSCO Metazoa genes in this assembly (Table 1). In fact, 26.9% of the corresponding genes in GA1 were reported to be duplicated, whereas only 0.3% were considered duplicated in GA2. Specifically, out of 257 BUSCO Metazoa genes that occurred multiple times in GA1, 256 were duplicated. Of these, 252 occurred once each in GA2. In GA1, the corresponding gene pairs resided on contigs with markedly different multiplicities, whereby the median for high-multiplicity contigs (132 x) was about nine times higher than that for the low-multiplicity contigs (15 x). We observed the same ratio in all high and low multiplicity contigs of GA1 (see below). Furthermore, the direction of the ratio was the same in mitochondrial haplotypes B and A (see above). Thus, *S. nebaliae* specimens carrying mitochondrial haplotypes A and B were most probably identical with carriers of nuclear genomes A and B, respectively.

### Results of read depth analysis is consistent with lineage formation in *S. nebaliae*

Analysis of k-mers provided further evidence for distinct lineages in *S. nebaliae*. In particular, k-mers that GenomeScope v. 1.0 classified as putative errors (1.3%) should partially have originated from less abundant carriers of nuclear genome A (Fig. 4: light blue fill to the left of the red curve). In turn, k-mers to the right of the red curve in Fig. 4 should primarily reflect more abundant DNA of *S. nebaliae* specimens that carried nuclear genome B. Based on B k-mers, GenomeScope estimated haploid genome size to be in the range of 45.7 Mb, with non-repetitive (unique) and duplicated portions of 81.3 and 2.1%, respectively. Probably due to genetic variation within B carriers, heterozygous k-mers peaked at higher multiplicity (coverage in GenomeScope terminology) than in the model graph for a diploid organism. In contrast, the larger peaks giving homozygous portions were at very similar multiplicity values in k-mers representing genome B and the model curve (for interpretation, see [42, 43]). The smaller peak of the blue-fill area in a range of ca. 200–250 x most likely indicated the duplicated portion in the nuclear genome of *S. nebaliae*.

Back-mapping of the gDNA reads used for assembling the contigs confirmed the intended pattern (Fig. 5). Thus, the graph for GA1 (dashed line) showed peaks in the low-multiplicity range (7 x and 15 x) which most likely corresponded to the heterozygous and homozygous portions in nuclear genome A in addition to foreign DNA. Two additional peaks at higher multiplicity values (81 x and 131 x) had smaller frequencies and probably represented genome B contigs. The corresponding distribution in GA2 (solid line) was confined to two stronger peaks in a higher multiplicity range. These should give the heterozygous and homozygous portions in nuclear genome B once more. Very similar multiplicities of these two peaks (79 x and 132 x) and their counterparts in GA1 (see above) reinforced that GA1 contained genome B contigs in addition to genome A contigs. Taking the homozygous peaks for both *S. nebaliae* genomes in GA1 as a reference, nuclear genome B (131 x) should have been nine times as abundant as genome A (15 x) in the in the DNA pool used for library preparation.

The previous pattern was reproduced in Merqury [44] analysis, where k-mers were mapped to the genome assemblies. Thus, GA1 and G2 shared k-mers peaking at higher multiplicity values of 72 x and 118 x (Fig. 6). These peaks corresponded to the ones at 77 x and 131 x in GenomeScope analysis (Fig. 4), thus representing heterozygous and homozygous proportions in the haploid nuclear genome of *S. nebaliae* genome B [42–44]. Slightly deviant multiplicity values for the two peaks in Merqury analysis in relation to GenomeScope analysis probably reflected differences in the settings. In particular, Merqury was run with a k of 18 whereas k was 21 in GenomeScope analysis. Either way, as in GenomeScope analysis (Fig. 4), the distribution for GA2 k-mers exhibited an additional smaller elevation at a multiplicity of 224 x, which should have represented the duplicated portion in the haploid nuclear genome of *S. nebaliae* (Fig. 6B). In contrast, the distribution for GA1 displayed a prominent k-mer peak at lower multiplicity (8 x), which in GenomeScope analysis was contained in the area left of the red line (Fig. 4).

### Small repetitive portion and only few SINEs in the nuclear genome of *S. nebaliae*

According to Repeatmasker, long interspersed nuclear elements (LINEs) made up the largest share of the repetitive portion in GA2 (4.5%), followed by unclassified repeats (4.3%), DNA elements (2.4%), long terminal repeats (LTR: 2.2%) and simple repeats (2.2%). Yet, only a few short interspersed nuclear elements (SINEs) were annotated (0.03%). Altogether, the repetitive portion in GA2 amounted to 16.2% or 7.1 Mb according to Repeatmasker (Additional file 1). Thus, ca. 83.8% of the *S. nebaliae* nuclear genome should be repeat-free. This is in accordance with the range of the repeat-free complement as estimated by GenomeScope (36.7–37.2 Mb), thus reinforcing the quality of GA2.

### Transcriptome metrics are in accordance with lineage formation and gene loss in *S. nebaliae*

Transcriptome assemblies 1 and 2 (TA1 and TA2) were highly congruent with the respective genome assemblies used for their guided reconstruction with Trinity [45]. For example, only five of the 34,859 TA2 transcripts could not be mapped with GMAP [46] to GA2. The median score of all mappings was 98 (out of 100), the average score was 93. In 96% of the cases, the mapping score was 80 or higher (Additional file 2). More importantly, different GC contents of both draft transcriptomes (TA1: 36.0%, TA2: 34.0%) were consistent with distinct *S. nebaliae* lineages again, as were additional metrics of both transcriptome assemblies (Table 3). In particular, TA2 contained about half as many transcripts of 200 bp or more (~ 35,000) than TA1 (~ 72,000). These were condensed to about half as many SuperTranscripts in TA2 (~ 23,000) than TA1 (~ 47,000). In addition, SuperTranscripts spanned about 27 Mb in TA2 and about double as much in TA1 (56 Mb). According to quotients of transcriptome and genome spans (TA1/GA1, TA2/GA2), 56.7 and 62.1% of GA1 and GA2, respectively, should be occupied by coding sequences. As to be expected for coding sequences, their GC contents were overall increased in relation to the genome assemblies they were residing in (Tables 1, 3). Not least, both transcriptome assemblies testified to marked gene loss in *S. nebaliae*. In fact, 34.3 and 35.2% of the screened BUSCO Metazoa genes were classified as missing in TA1 and TA2, respectively (Table 3). This was close to corresponding percentages in GA1 (35.4%) and GA2 (36.5%) as reported in Table 1.

**Table 3:** Characteristics of the *S. nebaliae* transcriptome

Metric	TA1 (GA1-guided) Genomes A + B	TA2 (GA2-guided) Genome B
Transcript number (minimum 200 bp)	72,072	34,859
Length of transcripts [bp]	97,940,784	45,737,361
Number of SuperTranscripts	47,106	23,214
Total length of SuperTranscripts [bp]	56,155,191	27,236,230
Mean length of SuperTranscripts [bp]	1192	1173
GC content of SuperTranscripts [%]	36.0	34.0
BUSCO – Metazoa genes* [%]	M = 34.3, D = 41.4	M = 35.2, D = 1.8

*GA1* nuclear genome assembly 1, *GA2* nuclear genome assembly 2, *D* duplicated BUSCOs, *M* missing BUSCO genes, *TA1* transcriptome assembly 1, *TA2* transcriptome assembly 2. Asterisks highlight references to BUSCO v. 4.0.6, database db10

**Fig. 8:**
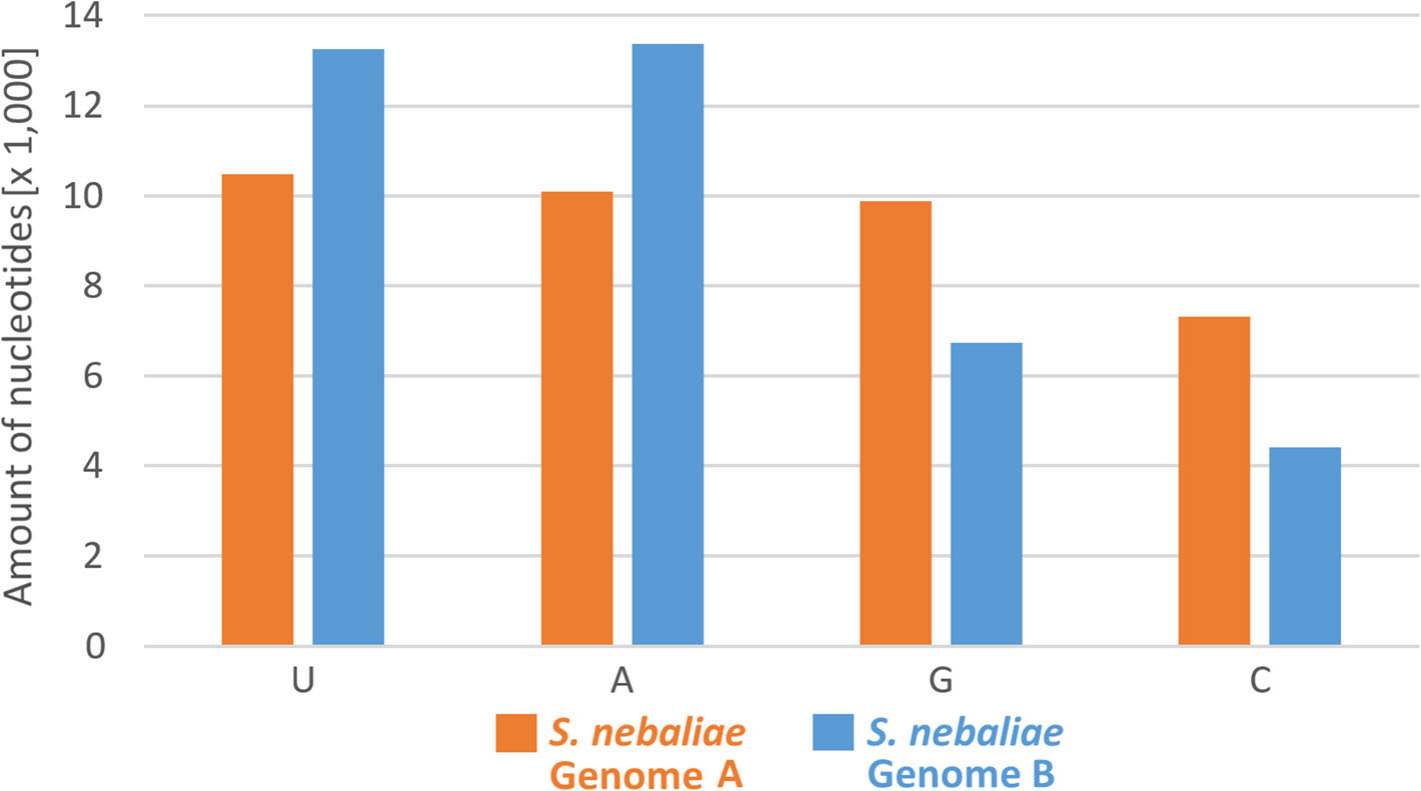
Differential nucleotide frequencies at third codon position in *S. nebaliae* genomes A and B. Absolute counts were inferred from 100 concatenated BUSCO Metazoa genes selected according to multiplicity ratios of the contigs they resided on in the range of 8–14 x. The AU skew was stronger in genome B than genome A

Similar to GA2 (0.3%), the proportion of duplicated BUSCO Metazoa genes was low in TA2 (1.8%) (Tables 1, 3). Recalling above evidence from frequency distributions, much higher proportions of “duplicated” genes in GA1 (26.9%) and TA1 (41.1%) presumably reflected the representation of gene pairs in nuclear genomes A and B. We took advantage of this fact and extracted cDNAs of the presumed gene pairs of genomes A and B, which BUSCO had classified as duplicated, from GA1. Of these, we retained 100 gene pairs residing on contigs with read depth differences in the range of 8–14 x, which corresponded to slightly relaxed demands when compared to the multiplicity ratios in mitochondrial haplotypes and genomic contigs (see above). As another precondition for further consideration of a gene pair, orthologues had to occur in all the species used for downstream comparisons. Plotting GC contents of the *S. nebaliae* contigs, which fulfilled these criteria, against their individual multiplicity values supported the genetic distinctness of genomes A and B. As in the aforementioned genome and transcriptome comparisons, the GC content was higher in the selection of contigs carrying BUSCO Metazoa genes representing genome A (mean = 36.2%, median = 36.1%) than in their counterparts representing genome B (mean = 31.5%, median = 31.2%) (Fig. 7). The difference was significant according to Mann-Whitney U (MWU) test (FDR = 0.000). In addition, the coding regions of the 100 BUSCO Metazoa genes analyzed exhibited differential codon usage at the third position. In particular, the skew toward AU into prospective *S. nebaliae* genome B was much more prominent than in presumed genome A (Fig. 8).

Taking the different lines of evidence (mitochondrial haplotypes and nuclear genomes with equidirectional ratios of abundancies, differential GC content, percentages of duplicated BUSCO genes, differential codon usage etc.), we consider the variation observed as indicative of strain formation in *S. nebaliae*. Thus, we will collectively refer to *S. nebaliae* specimens carrying mitochondrial haplotype A and nuclear genome A as to strain A. Carriers of mitochondrial haplotype B and nuclear genome B will be designated strain B henceforth.

### Divergent gene compactness within Rotifera-Acanthocephala

**Fig. 9:**
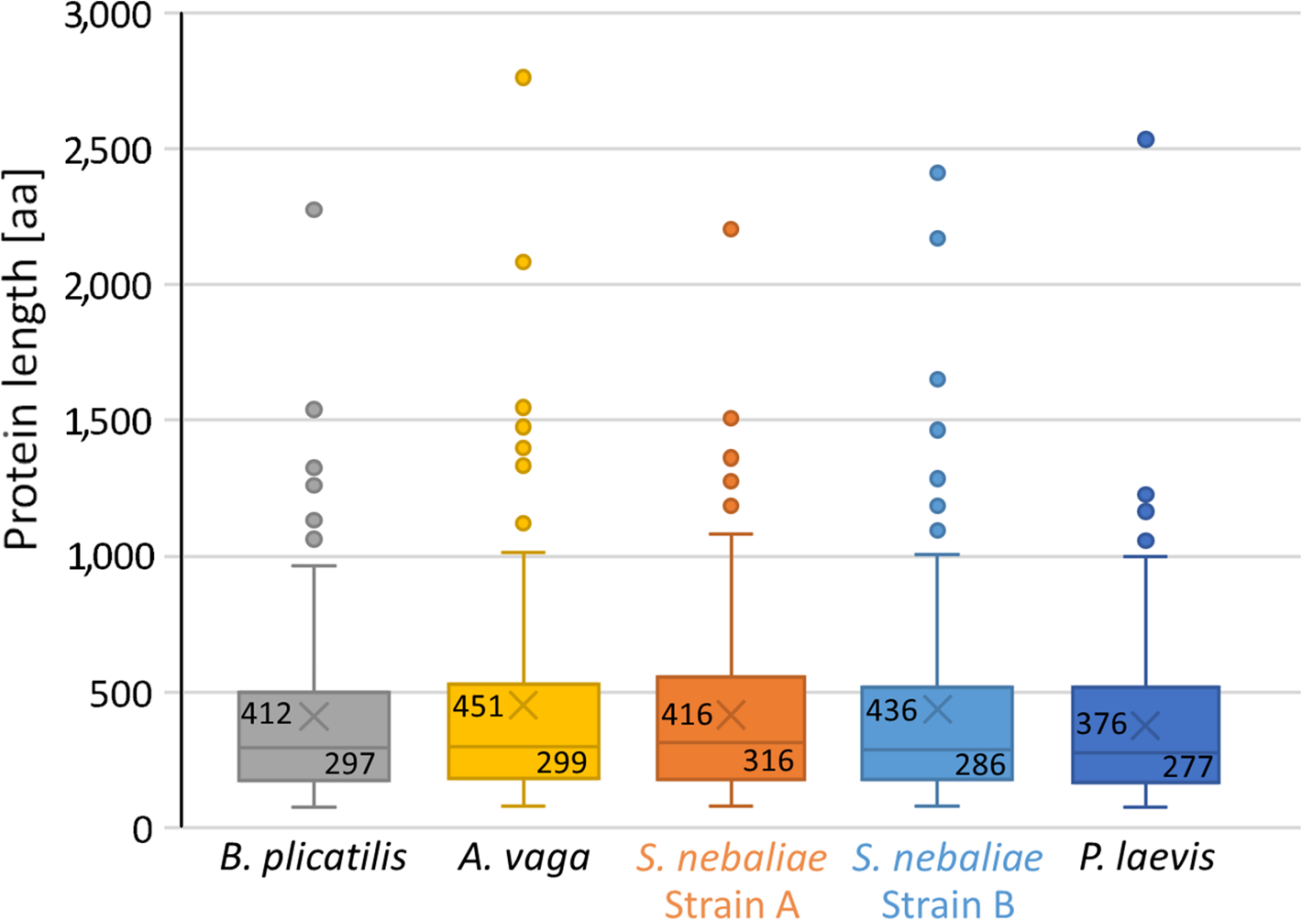
Distributions of protein lengths within the Rotifera-Acanthocephala clade. Protein length is given in amino acids (aa). High similarity of distributions is consistent with the orthology of the 100 BUSCO Metazoa proteins compared (FDR > 0.05; KW test). Boxes symbolize the aa range of the middle 50% of proteins. Within boxes, upper values represent mean values (marked with a light-grey X in each case) and lower values medians (horizontal lines). Upper whiskers correspond to 1.5 x interquartile ranges (IQRs). Dots give single proteins with lengths exceeding IQRs. Lower whiskers extend to minima

**Fig. 10:**
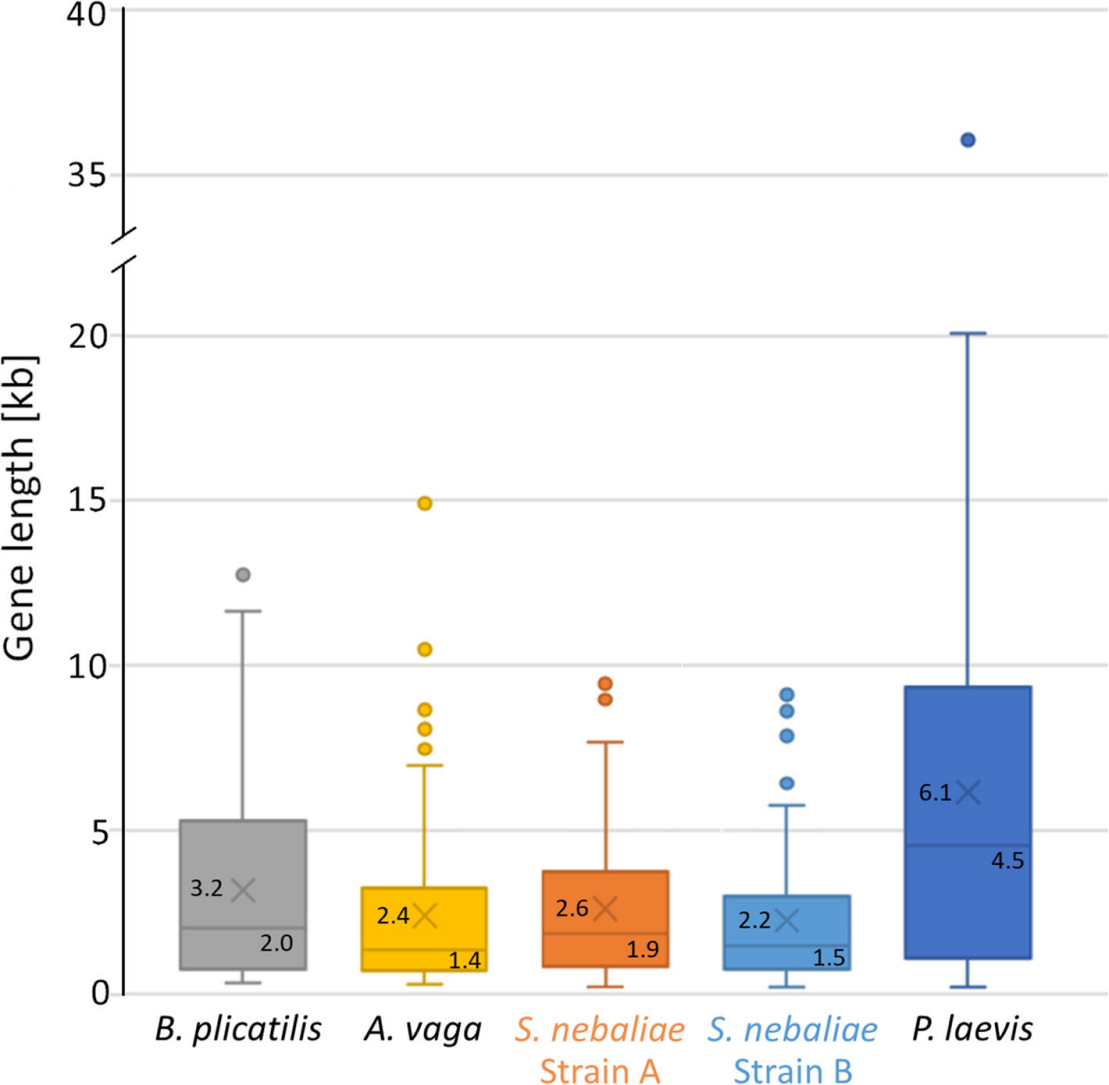
Length of protein-coding genes within the Rotifera-Acanthocephala clade. Gene length is given in kilobases (kb). According spans have been derived from annotations for 100 orthologous BUSCO Metazoa genes. Levels of gene length significantly differ across the five taxa included (FDR = 0.000; KW test). Because the encoded proteins were similar in length (see Fig. 9), the differences shown here testify to divergent spans of intronic segments. Thus, genes are least compact in *P. laevis*, followed by *B. plicatilis*, while genes were most compact in *A. vaga* and both *S. nebaliae* strains. Within the boxes, upper values represent mean values (marked with X in each case) and lower values medians (horizontal lines). Upper whiskers correspond to 1.5 x interquartile ranges (IQRs). Dots give single proteins with lengths exceeding IQRs. Lower whiskers extend to minima

We compared the aforementioned 100 BUSCO Metazoa genes of *S. nebaliae* strains A and B with their orthologues in the monogonont *Brachionus plicatilis*, bdelloid *A. vaga*, and acanthocephalan *P. laevis*. In support of the orthology of the proteins encoded, levels of protein length did not differ significantly across the five taxa studied according to Kruskal-Wallis (KW) test (FDR > 0.05; Fig. 9). In contrast, levels of gene length as derived from BUSCO annotations were significantly different across the five taxa (FDR = 0.000; KW test). Thereby, length distributions were most similar in both *S. nebaliae* strains and *A. vaga*, whereas gene annotations had overall larger spans in *B. plicatilis* and *P. laevis* (Fig. 10). Recalling similar levels of protein length, differences in gene length must have reflected divergent intronic spans. Thus, genes were the least compact in *P. laevis*, followed by *B. plicatilis*, while gene compactness was highest in *A. vaga* and both *S. nebaliae* strains.

### Gene repertoire size in epizoic *S. nebaliae* ranges between corresponding numbers for (other) wheel animals and the acanthocephalan

Genome assembly 2 contained 606 BUSCO Metazoa genes classified as single copy, duplicated and fragmented. This was less than identified in the draft genomes for the monogonont *B. plicatilis* (863) and the bdelloid *A. vaga* (855). Still, the number of recognized genes in *S. nebaliae* was higher than in the acanthocephalan *P. laevis* (440). Missing BUSCO Metazoa genes showed the expected inverse order, with 91 in *B. plicatilis*, 99 in *A. vaga*, 348 in *S. nebaliae* strain B and 514 in *P. laevis*. In addition, the degenerate tetraploid *A. vaga* [20] had the fewest complete single copy (178) and by far the most duplicated BUSCO Metazoa genes (662). On the contrary, there were almost exclusively single copy genes in the other three genome assemblies. For *S. nebaliae*, this pattern was consistent with above results from k-mer analyses, according to which the duplicated portion of GA2 was small (Figs. 4, 6B).

Since genes classified as missing might actually have been overlooked due high divergence, we repeated the search for BUSCO Metazoa genes with the training option of the algorithm enabled. These additional analyses were confined to *S. nebaliae* (GA2) and *P. laevis*, since numbers of BUSCO Metazoa genes for the monogonont and the bdelloid already exceeded 90% without training. However, the second run did not result in a substantial increase in the number of BUSCO Metazoa genes detected. In fact, the training resulted in very similar percentages of missing genes (*S. nebaliae*: 35.5%, *P. laevis*: 52.3%) when compared to the values reported in Table 4 (*S. nebaliae*: 36.5%, *P. laevis*: 53.9%). This prompted us to additionally screen the *S. nebaliae* and *P. laevis* draft genomes for BUSCO Eukaryota genes, which are commonly regarded as being more conserved than metazoan ones. Although this approach uncovered relatively more genes, the general pattern was reproduced. Thus, the percentage of missing genes remained considerable in the *S. nebaliae* draft genome (GA2: 21.1%) and still was increased in the *P. laevis* draft genome (31.5%).

**Table 4:** Comparative analysis of gene repertoires within Rotifera-Acanthocephala

Metrics of BUSCO Metazoa genes^**a**^	***B. plicatilis***	***A. vaga***	***S. nebaliae*** Strain B^**b**^	***P. laevis***
Number of complete single copy genes	843	178	566	343
Percentage of complete single-copy genes [%]	88.4	18.7	59.3	36.0
Number of duplicated genes	8	662	3	33
Percentage of duplicated genes [%]	0.8	69.4	0.3	3.5
Number of fragmented genes	12	15	37	64
Percentage of fragmented genes [%]	1.3	1.6	3.9	6.7
Sum of BUSCO genes	863	855	606	440
Number of missing genes	91	99	348	514
Percentage of missing genes [%]	9.5	10.3	36.5	53.9

Datasets analyzed: GCA_010279815.1 (*B. plicatilis*), GCA_000513175.1 (*A. vaga*), present GA2 (*S. nebaliae* strain B), GCA_012934845.1 (*P. laevis*). ^a^BUSCO v. 4.0.6, database odb10; ^b^Percentages of duplicated and missing genes are identical to corresponding entries in Table 1

**Fig. 11:**
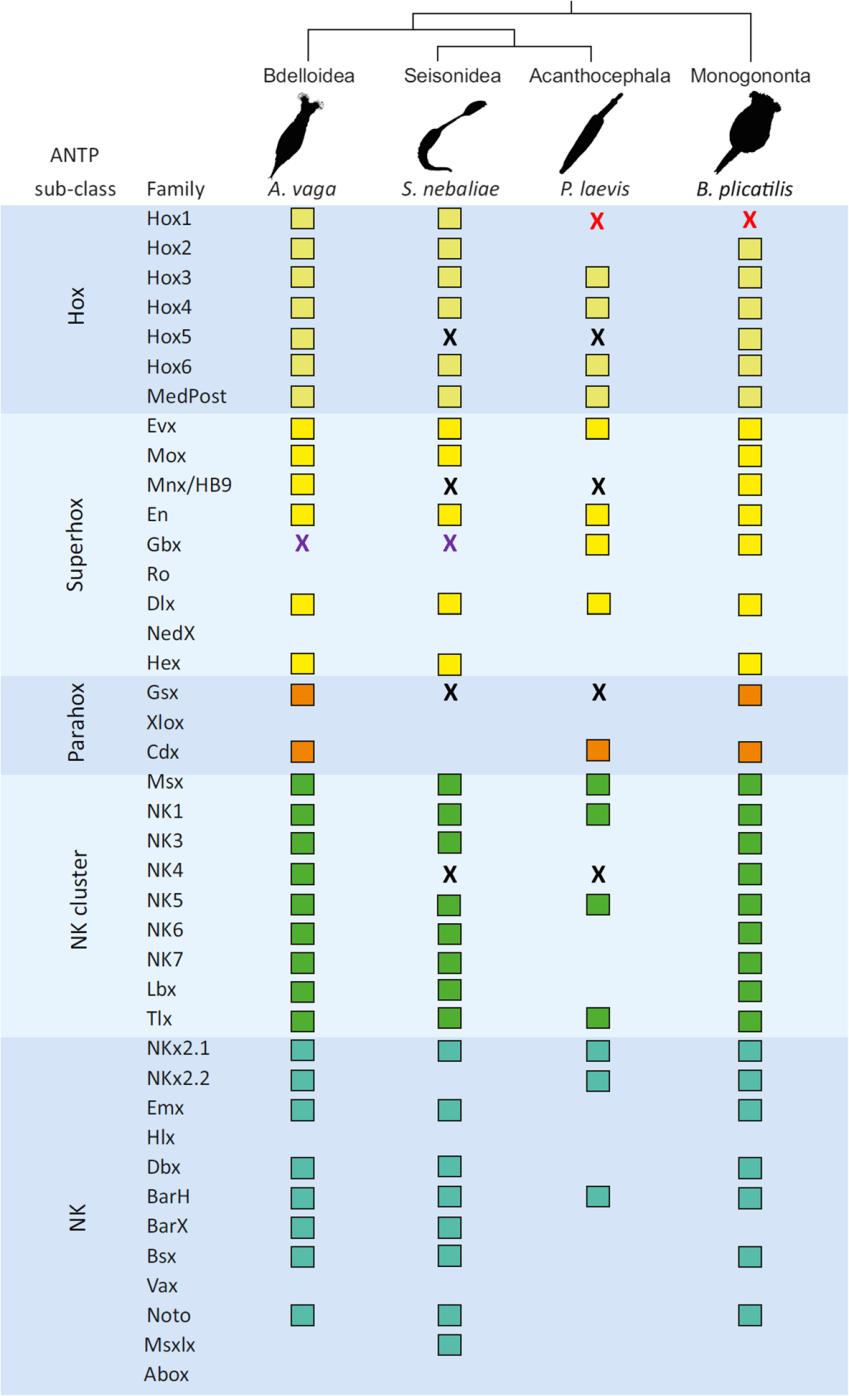
Representation of ANTP-type gene families within Rotifera-Acanthocephala. Colored boxes indicate that genes of particular gene families were identified. Absence of a box means that no corresponding gene was found. Missing gene families in species pairs are highlighted by x’s (black for Seisonidea-Acanthocephala; red for Monogononta-Acanthocephala, lilac for Bdelloidea-Seisonidea). Out of the 40 gene families searched by TBLASTN, 35 were determined in at least one of the draft genomes analyzed. Their presence/absence indicates an intermediate size of the corresponding complement in *S. nebaliae* (GA2) between the monogonont (*B. plicatilis*) and the bdelloid (*A. vaga*) on the one hand, and the acanthocephalan (*P. laevis*) on the other. Shared absence of *hox 5*, *mnx/hb9*, *gsx* and *nk4* gene families in the draft genomes of *S. nebaliae* and *P. laevis* would be in line with a monophyletic origin of Seisonidea and Acanthocephala (Pararotatoria) as visualized by the tree at the upper margin. Note that there is no lacking pair of gene families in support of monophyletic Lemniscea (Bdelloidea+Acanthocephala). Also, there is no missing ANTP-like gene family in support of monophyletic Eurotatoria (Monogononta+Bdelloidea)

In a complementary approach, we checked for the presence of genes coding for transcription factors with ANTP-type homeodomains (Fig. 11). Corresponding screens failed to determine any member of five ANTP-type gene families (*ro*, *nedX*, *xlox*, *hlx* and *abox*) in the monogonont *B. plicatilis*, the bdelloid *A. vaga*, the seisonid *S. nebaliae* strain B (GA2) and the acanthocephalan *P. laevis*. The remaining 35 gene families searched occurred in at least one of the four draft genomes analyzed. Their presence-absence patterns yielded additional hints for an intermediate comprehensiveness of the seisonid gene repertoire, as observed before in BUSCO Metazoa genes. Thus, out of the aforementioned 35 ANTP-type gene families, *B. plicatilis* and *A. vaga* lacked four and two, respectively. With 18, more than half of the 35 ANTP-type gene families appeared to be absent in the draft genome of the acanthocephalan *P. laevis*. With eight ANTP-type gene families, for which no member was found, GA2 occupied the anticipated intermediate position once more. Furthermore, the draft genomes of *S. nebaliae* and *P. laevis* consistently lacked evidence of *hox 5*, *mnx/hb9*, *gsx* and *nk4* (see black x’s in Fig. 11). In turn, there was no exclusive absence of any ANTP-type gene family in the bdelloid and acanthocephalan draft genomes.

### Phylogenomics: rooting with the LCA places Seisonidea as sister to Acanthocephala

**Fig. 12:**
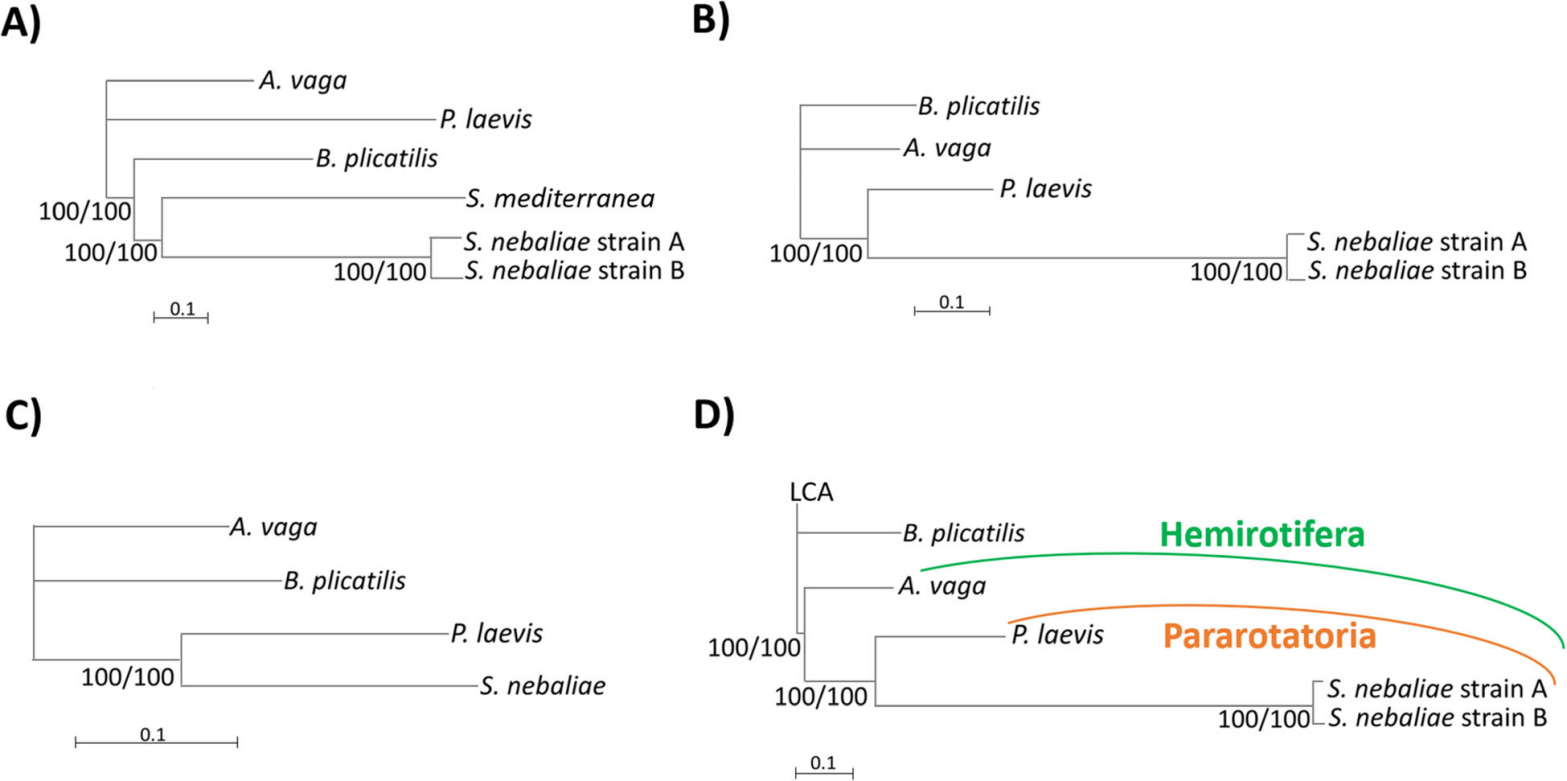
Phylogenetic relationships within Rotifera-Acanthocephala. **A)** Unrooted BI tree as inferred from concatenated sequences of 100 orthologous metazoan proteins (dataset: Concat100-6OTUs). Rooting the tree with the platyhelminth *S. mediterranea* would invert the topology, thus suggesting a monophyletic origin of *A. vaga* and *P. laevis* in support of Lemniscea (Bdelloidea+Acanthocephala). **B)** Unrooted BI tree built from an alignment devoid of singleton positions and lacking the platyhelminth (Concat100-noSing-5OTUs). Here, *S. nebaliae* clusters with *P. laevis*. **C)** Unrooted BI tree after deletion of singleton positions, the outgroup representative and *S. nebaliae* strain B from the alignment (Concat100-noSing-4OTUs-A): The clustering of *S. nebaliae* and *P. laevis* is stable also when branch lengths are homogenized. The corresponding tree for Concat100-noSing-4OTUs-B showed the same topology. **D)** Rooted BI tree as derived from an alignment in which the platyhelminth sequence was replaced by a reconstructed sequence of the last common ancestor (LCA) of Rotifera-Acanthocephala (Concat100-6OTUs-LCA): Support for monophyletic Pararotatoria and Hemirotifera was maximal. Phylograms depicted were inferred with MrBayes v. 3.2.7a. Average deviation of split frequencies was always smaller than 0.01 in BI. Tree reconstruction with PhyML v. 3.3 led to identical topologies. Support values preceding slashes represent clade credibility values after discard of the first 25% of generations collected. Support values following slashes refer to the SH-like approximate likelihood ratio test (aLRT) in PhyML. The model of best-fit (LG + G + I + F) was determined by smart model selection (SMS)

*Seison nebaliae* clustered with the platyhelminth *Schmidtea mediterranea* in unrooted BI and ML trees inferred from an alignment containing translated sequences of the above 100 BUSCO Metazoa genes (Concat100-6OTUs; Fig. 12A). In contrast, *S. nebaliae* grouped together with the acanthocephalan *P. laevis* in additional BI and ML trees, which we had derived from edited alignments. In particular, the alternative topology was reflected in unrooted ML and BI trees built from an alignment devoid of singleton positions and lacking the platyhelminth (Concat100-noSing-5OTUs; Fig. 12B). However, the branch to *S. nebaliae* was disproportionately long because amino acid states unique to *S. nebaliae* but shared by both strains were not recognized as singletons by the script used for editing the alignment. To account for this limitation, we additionally generated alignments, which were pruned from singleton positions and lacked either *S. nebaliae* strain A or B in addition to the platyhelminth (Concat100-noSing-4OTUs-A; Concat100-noSing-4OTUS-B). This enabled that character states unique to *S. nebaliae* were recognized as such, followed by removal of the corresponding alignment positions. Notably, branch lengths were homogenized in the resulting trees and, regardless of the strain kept, *S. nebaliae* clustered with *P. laevis* (Fig. 12C; Additional file 1). Support for the internal branch separating the seisonid-acanthocephalan cluster and the monogonont-bdelloid cluster reached the maximum value in all corresponding BI and ML trees (for a tabular summary, see Table S3 in Additional file 1). Collectively, these results suggested that the long seisonid branch in the tree derived from the unedited Concat100 alignment was drawn to the long outgroup branch (Fig. 12A). To avoid this, we inferred the Concat100 sequence for the last common ancestor (LCA) of Rotifera-Acanthocephala. This was done with an intree assuming a star-like topology for Rotifera-Acanthocephala. Thus, no particular phylogenetic hypothesis was implied. Prior to subsequent tree reconstruction, we replaced the platyhelminth by the LCA sequence (Concat100-6OTUs-LCA). Corresponding BI and ML trees consistently yielded maximum support for monophyletic Pararotatoria (Seisonidea+Acanthocephala) within maximally supported Hemirotifera (Bdelloidea+Pararotatoria) (Fig. 12D). The tree additionally implied paraphyly of Eurotatoria (Monogononta+Bdelloidea) and Lemniscea (Bdelloidea+Acanthocephala).

## Discussion

### Two strains of *S. nebaliae* in the tidal flats off Roscoff

Based on DNA-Seq data for which DNA had been extracted from a pool of *S. nebaliae* specimens, we reconstructed two mitochondrial genome sequences (Fig. 1). One of the two was almost identical with a previously published mitogenome sequence of *S. nebaliae* [24], while the second one was novel. Both sequences exhibited considerably low sequence identity (82.5%) and markedly different lengths (strain A: 15.1 kb; strain B: 16.2 kb). Nevertheless, their sizes remained within the range of 13.6–16.8 kb reported for other members of the Rotifera-Acanthocephala clade [40, 47, 48] and phylogenetic analyses confirmed their origin in *S. nebaliae* (Fig. 3; Additional file 1). In addition, both mitogenomes showed the same gene order, which is unlikely to be coincidental [49]. As transcriptional analyses were supportive of their functionality (Fig. 2), we refer to these mitochondial genomes in *S. nebaliae* as to haplotypes A and B. Since the almost complete assembly of the haploid nuclear genome of *S. nebaliae* (GA2: ca. 44 Mb) would fit approximately twice into a second assembly reconstructed under relaxed conditions (GA1: ca. 99 Mb), a total of two nuclear genomes should have been represented in the sequenced DNA(s). In line with this, we found evidence of variation between the presumed nuclear genomes in *S. nebaliae*, in terms of GC content, codon usage, and gene compactness (Figs. 7, 8, 9 and 10). Distributions of k-mer frequency and multiplicity provided additional hints for the presence of genome variants in *S. nebaliae* (Figs. 4, 5 and 6). Thereby, a common thread was that both the mitogenome and the nuclear genome were represented in a clearly more (B) and a less abundant variant (A). This consistency is hardly compatible with the occurrence of both mitochondrial haplotypes in single individuals of *S. nebaliae*, as reported for other Metazoa (e.g., [50]). Rather, the carriers of mitochondrial haplotypes A and B should be identical with specimens having genome variants A and B, respectively. Thus, there should be genetically more or less separated lineages within *S. nebaliae* in the tidal flats off Roscoff, France.

The degree of mitochondrial sequence dissimilarity (17.5%) would also justify the postulation of separate species, as done in other cases based on smaller values (e.g., [51]). Cryptic speciation in seisonids would fit well into the general picture of Rotifera-Acanthocephala, considering corresponding reports for Monogononta, Bdelloidea and Acanthocephala [32–34]. On the other hand, molecular features such as low mtDNA identity alone are not necessarily sufficient for species delimitation [52, 53] and revision of the *Seison* taxonomy seems premature to us as long as potential morphological or ecological specificities of both strains/species have not been checked for. For example, the lineages might differ in their spatial and nutritional niches on the common host *N. bipes* as it is commonly assumed for *S. nebaliae* and *P. annulatus* [4]. Both *S. nebaliae* lineages might also show differential population dynamics in dependence of seasonal temperatures as described for sessile rotifers before [54]. As these questions have not yet been adressed, we leave it at the distinction of *S. nebaliae* strains in the present study.

### Consistent genome and transcriptome metrics in *S. nebaliae* and other members of Rotifera-Acanthocephala

The GC content found for *S. nebaliae* was within the previously known boundaries for Rotifera-Acanthocephala. However, a closer look shows that the GC content in the seisonid reference genome (GA2: 31.9%) was first of all very similar to identical to draft genomes of an acanthocephalan (*P. laevis*: 32.9%) and several bdelloids (*A. vaga*: 31.2%, *A. ricciae*: 35.6%, *R. magnacalcarata*: 31.9%, *R. macrura*: 32.6%) [21, 23]. Overall, their values were markedly higher than for the draft genomes of the monogononts *B. calyciflorus* (24.2%) and *B. plicatilis* (26.4%) [55, 56]. In light of the tree shown in Fig. 12D, the nuclear genome of the LCA of Hemirotifera might thus have had a GC content of ca. 30% or higher. In addition, the total number of nuclear genes in seisonids should be within the range of ca. 11,000 to ca. 69,000 as reported for other members of the Rotifera-Acanthocephala clade [21, 23, 55–58]. The number of SuperTranscripts inferred here for *S. nebaliae* (ca. 23,000) at least does not contradict such a vague estimation (Tables 1, 3). 

### Measures counteracting attraction to the root place the seisonid next to the acanthocephalan branch

The question remains why Seisonidea clustered with Acanthocephala in part of the previous molecular analyses (e.g., [18, 19]; see also Supplementary Fig. S1 to [59]), but otherwise branched basally within Rotifera-Acanthocephala. Present ML and BI based tree reconstructions add to previous hints that misleading signal could be the reason for a basal branching of Seisonidea [24]. Indeed, the seisonid branch was the longest in trees in which the lineage diverged basally [38, 59]. However, it is getting increasingly clear that attraction of a long ingroup branch to the outgroup or root leads to systematic error in phylogenetic analyses (e.g., [60]). We found a strong indication of this phenomenon in the unrooted tree derived from 100 concatenated BUSCO Metazoa genes (Fig. 12A): Here, *S. nebaliae* grouped together with the outgroup representative *S. mediterranea*. Rooting the tree with the latter would hence result in a basal branching of *S. nebaliae*. But after implementation of measures preventing LBA between the platyhelminth and *S. nebaliae*, the latter clustered with *P. laevis* (Fig. 12B,C). A requirement for the rearrangement of the tree topology in analyses of the edited datasets was the combined removal of the platyhelminth and of singletons (see Additional file 1 for a detailed discussion). At this point, it remains to be noted that editing of the initial dataset provided clues according to which artificial signal might be the reason for a basal branching of *S. nebaliae*. A significant part of the problem is likely to be attraction to the outgroup.

Given hundreds of millions of years that will have elapsed elapsed since the split of Rotifera-Acanthocephala and any other lineage with extant species (reviewed in [9]), outgroups will always be highly divergent. Thus, attraction of a particular ingroup branch to the root would almost certainly persist in a wide range of outgroup species. In fact, corresponding results of other studies can be interpreted in this sense [38, 59]. Against this background, we consider rooting via a reconstructed ancestral sequence as a reasonable way to address the problematic resolution of deep splits. In the present study, the approach stably led to monophyletic Pararotatoria (Seisonidea+Acanthocephala) within monophyletic Hemirotifera (Bdelloidea+Pararotatoria) (Fig. 12D; Additional file 1 [19, 24, 37]). This adds to previous findings that Eurotatoria (Monogononta+Bdelloidea) is probably a paraphyletic assemblage [14, 38, 61–63]. Furthermore, Lemniscea would be paraphyletic according to the BI tree topology in Fig. 12D and the corresponding ML tree in Additional file 1. Actually, it would not even be possible to root the BI trees in present Fig. 12B, C and their ML counterparts in a way that monophyletic Lemniscea emerge. There also seems to be no morphological feature in support of monophyletic Lemniscea. In particular, the eponymous lemniscs, neck-based invaginations of the syncytial tegument in Acanthocephala, do not correspond to cellular hypodermal cushions supporting the wheel organ in Bdelloidea [64]. In contrast, particular filament bundles in the tegument and two rows of electron-dense bodies alongside the sperm flagellum could be evolutionary novelties shared by Seisonidea and Acanthocephala [4, 11–13, 36]. Not least, mitochondrial gene order was shown to be in line with monophyly of Pararotatoria and Hemirotifera [24].

### Genome evolution within Rotifera-Acanthocephala

The likely phylogenetic relationships depicted in Fig. 12D additionally implicate that the LCA of Rotifera-Acanthocephala may have passed a genome of about 50 Mb, as occurring inside Monogononta [22], to the descendant lineages of Hemirotifera and Pararotatoria. Thus, a smaller genome like in *S. nebaliae* (GA2: ca. 46 Mb) would have resulted from reduction as it appears to be reflected in the gene repertoire as well (see below). In turn, size increases should have independently occurred in the evolution of Bdelloidea, Acanthocephala and some Monogononta. Thus, within Monogononta, larger genome size has been associated with the spread of transposable elements [22]. In Bdelloidea, genome duplication laid the basis for greater genomes although subsequent losses of genome parts obscure the original pattern of tetraploidy [20, 21, 65–67]. In line with this, we found the percentage of duplicated BUSCO Metazoa genes increased in the bdelloid *A. vaga*, whereas duplicated BUSCO genes were rare in the drafts for acanthocephalan, monogonont and seisonid genomes (Table 4). Lastly, genome size as reported for the acanthocephalan *P. laevis* is likely to result from multiplication of a wide variety of repeat types [23] in combination with a decrease in gene compactness (present study). But even if genome size increases reflect the spread of transposable elements, SINEs should have been of minor importance in the different lineages of Rotifera-Acanthocephala. In fact, SINEs appear generally to be little represented in rotiferan and acanthocephalan draft genomes available so far [21–23, 55].

### Increasing gene loss with growing ties to the host

We cannot exclude that individual genes were missing in the draft genomes, although they are present in a given species. On the other hand, the possibility that missing BUSCO Metazoa genes are partially located in the unassembled regions should not be limited to *S. nebaliae* and *P. laevis*. But if incompleteness of the assemblies played a role at all, such an effect should not be substantial. Thus, the total size of the *P. laevis* haploid genome draft of approximately 260 Mb was very close to the GenomeScope prediction of 265–281 Mb [23]. Similarly, GenomeScope estimated the size of the nuclear genome of *S. nebaliae* to be approximately 46 Mb, of which 44 Mb could be delivered in assembly GA2. Consistent with this, Merqury for GA2 calculated a k-mer completeness of approximately 92% (Table 2). However, a maximum of 8% of the genomes awaiting reconstruction in each of the two species is unlikely to explain the failure to determine substantial proportions of BUSCO Metazoa genes as reported in current Table 4. It remains possible that genes have not been detected because the genomes are more derived, especially in *S. nebaliae* and *P. laevis*. In agreement with this, we determined BUSCO Eukaryota genes, which are usually more conserved than BUSCO Metazoa genes, to larger proportions in the draft genomes of *S. nebaliae* and *P. laevis*. Nevertheless, the overall picture of more missing genes in the *P. laevis* than *S. nebaliae* draft genomes remained unchanged in BUSCO Eukaryota genes. Ultimately, the same picture seems to emerge in presence-absence analyses of ANTP-type gene families (Fig. 11). Still, it should be emphasized that we do not claim that the numbers of missing genes as reported by us are defintive. However, we consider valid the basic pattern that, under the same settings, the repertoires of discovered genes decrease from free-living rotifers via epizoic seisonids to endoparasitic acanthocephalans. This may be due to the increasing outsourcing of functions with growing ties to a host. 

Outsourcing of functions and associated gene loss is well known for parasites (e.g., [68]). However, the close correspondence with the probable phylogenetic relationships within Rotifera-Acanthocephala is, in our view, striking. Thus, seisonids use leptostracan crustaceans as a substrate, probably also benefiting from their food as commensals. They may even feed on hemolymph depending on the seisonid species [1–3, 26]. Yet, living on a host should require less functional autonomy than living without host association such as in the monogonont *B. plicatilis* and the bdelloid *A. vaga* (compare [4]). An epizoic lifestyle as in extant seisonids is also probable for the LCA of Pararotatoria [18, 24].

Despite the preliminary nature of the results, it seems worthwhile mentioning that the ANTP-type gene families, for which no member was determined in the draft genomes for *S. nebaliae* and *P. laevis* (*hox5*, *mnx/hb9*, *gsx* and *nk4*), steer nervous system development in other taxa [69–72]. This does not have to be their only function, as the *hox* gene cluster has primarily been implicated in anterioposterior patterning [73, 74]. Regardless, the absence of all or part of the four gene families could indicate a simplified development of the nervous system in the pararotatorian LCA compared to free-living monogononts and bdelloids. Extending this argument, the low number of ANTP-type and other genes in *P. laevis* (Fig. 11) could indicate further reduced functional autonomy in endoparasitic acanthocephalans. In fact, thorny-headed worms are commonly considered to be simplified metazoans [75]. Acanthocephalans such as *P. laevis*, for example, lack excretory organs and none of the extant species has a digestive tract (e.g., [9, 76]). At least it is not contradictory that two genes, *hox1* and *hox2*, which are thought to play a role in the development of the stomatogastric nervous system [73, 74], could not be determined in the draft genome of *P. laevis* [23].

## Conclusion

Present analyses suggest co-existence of two *S. nebaliae* strains on *Nebalia bipes* off the French Channel coast. Their mitogenomes (Fig. 1) differed considerably in terms of (NCR) length and sequence identity (ca. 82%). As shown by phylogenetic analyses, both mitochondrial genomes represented *S. nebaliae.* Thus, none of the mitochondrial sequences was derived from the crustacean host or from *P. annulatus,* the other seisonid species living on opossum shrimps (Fig. 3). Additional validation steps corroborated transcription and hence functionality for both *S. nebaliae* mitogenomes. Hence, none of these should represent a nuclear mitochondrial integration (Fig. 2). Therefore, we consider it appropriate to refer to the reconstructed mitochondrial genomes as to haplotypes (A and B) within *S. nebaliae*. We also found evidence for two nuclear genomes (A and B) that differed in gene compactness, GC content and codon usage (Figs. 7, 8, 9 and 10). Since the mitochondiral and nuclear genomes exhibited equidirectional abundance ratios, it is reasonable to assume that genetically distinct strains (A and B) exist in *S. nebaliae*. In both strains, haploid nuclear genome size should be ca. 46 Mb, of which ca. 44 Mb were contained in assembly GA2. Thus, the seisonid nuclear genome could be the smallest within Rotifera-Acanthocephala. Its size might reflect a reduction, provided haploid genome size was about 50 Mb in the LCA of Rotifera-Acanthocephala. Under the same premise, larger genomes should have evolved independently within Monogononta and in the stem lines of Bdelloidea and Acanthocephala [20, 22, 23]. Additional genome and transcriptome metrics for *S. nebaliae* were within the ranges in other members of Rotifera-Acanthocephala. In particular, the number of BUSCO Metazoa genes determined in the draft genome of *S. nebaliae* was intermediate between corresponding counts for free-living monogonont and bdelloid wheel animals and an endoparasitic acanthocephalan (Fig. 11; Table 4).

According to phylogenomic analyses of 100 proteins encoded by concatenated BUSCO Metazoa genes, a basal branching of Seisonidea within the Rotifera-Acanthocephala clade is primarily due to LBA to the outgroup or root. Indeed, measures avoiding such an effect led to a clustering of *S. nebaliae* with the acanthocephalan representative. In particular, rooting the tree via an ancestral sequence resulted in maximum support for monophyletic Pararotatoria (Seisonidea+Acanthocephala) within monophyletic Hemirotifera (Bdelloidea+Pararotatoria) (Fig. 12). Missing evidence can always be due to non-finding. Nevertheless, it is worthwhile noting that the exclusive non-determination of members of four ANTP-type gene families in the draft genomes of *S. nebaliae* and the acanthocephalan *P. laevis* at least does not contradict the Pararotatoria hypothesis. In contrast, none of the ANTP-type gene families searched for lacked members in draft genomes of the bdelloid *A. vaga* and *P. laevis* only (Fig. 11). Thus, Lemniscea (Bdelloidea+Acanthocephala) [39] gained no support from presence-absence analysis of ANTP-type gene families.

Mapping lifestyles on the tree in Fig. 12D, a parsimonious scenario would be that seisonids have retained an epizoic lifestyle on jawed arthropods (Mandibulata) from their last common ancestor with acanthocephalans. Early acanthocephalans would then have entered the mandibulate host, followed by upward inclusion of jawed vertebrates (Gnathostomata) into the lifecycle (e.g. [9, 24]). When combined with results of BUSCO analyses, the corresponding tree suggests gradually increasing gene loss from the LCAs of Rotifera-Acanthocephala and Hemirotifera, both of which should have been free-living, to the probably epizoic LCA of Pararotatoria to the LCA of Acanthocephala, for which an endoparasitic lifestyle is almost certain (for presumed lifestyles in LCAs, see, e.g., [8, 9]). Ultimately, however, the finding that growing ties to the host associate with increasing gene loss inside Rotifera-Acanthocephala should hold true regardless of the phylogenetic hypothesis.

In addition to the above phylogenetic and evolutionary implications, the *S. nebalia* draft genome might proof useful for the development of a specific drug for the control of acanthocephalans, which turned out to be a pest in fish aquaculture (e.g., [77, 78]). Thus, it might be possible now to identify acanthocephalan genes or proteins as potential targets for a highly specific control of the parasites. Follow-up studies might further uncover subtle differences in population dynamics and/or morphology between the *S. nebaliae* strains determined herein (compare [35]). Future analyses might additionally clarify if *S. nebaliae* is a complex of cryptic species. Finally, the path shown here of rooting a tree via a reconstructed ancestral sequence instead of the sequence of an extant outgroup representative may generally prove useful in phylogenetic analyses of deeply splitting taxa.

## Methods

### Samples

Permit for collecting and processing opossum shrimps (Crustacea: Leptostraca: *N. bipes*) and their epifauna was given by European Marine Biological Resource Centre (EMBRC). Collections of opossum shrimps were carried out in spring 2016 and late summer 2019 at low tide from rock pools in the tidal flats off Roscoff (Brittany, France). The crustaceans were kept in a Dewar vessel and brought to the laboratory at the Station Biologique Roscoff, an institution of the EMBRC. Under the stereomicroscope, specimens of *S. nebaliae* and *P. annulatus* (Rotifera: Seisonidea, each) were separated from the shrimps and stored at − 80 °C. Isolation of nucleic acids followed in Mainz upon transportation of the samples on dry-ice.

### DNA sequencing and preliminary assembling

A pool of 594 *S. nebaliae* specimens (males and females) was digested using proteinase K, followed by DNA extraction with phenol-chloroform-isoamyl alcohol and precipitation in ethanol. The library prepared from this DNA was sequenced on four lanes on an Illumina NextSeq 500 platform (150 bp, paired-end mode, 74,690,183 pairs total). Quality of raw NGS data was checked with FastQC v. 0.11.9 (http://www.bioinformatics.babraham.ac.uk/projects/fastqc/), followed by trimming of Illumina adaptor sequences and quality trimming (HEADCROP:6 SLIDINGWINDOW:4:15 LEADING:3 TRAILING:3 MINLEN:20) with Trimmomatic v. 0.39 [79]. Processed reads were mapped against a masked human reference genome (HG19) with BBmap (https://sourceforge.net/projects/bbmap/), upon which sequences of potential human (*Homo sapiens*) origin were removed. In addition, DNA was isolated from a pool of 10 individuals of *P. annulatus* applying the same protocol. Following whole genome amplification (illustra GenomiPhi™ V2 DNA Amplification Kit) DNA was sequenced on one lane on an Illumina NextSeq 500 platform (150 bp, paired-end mode, 87,887,226 pairs total). Data quality assessment and trimming used the same programs as detailed above. Raw reads of both sequencing runs can be retrieved from EMBL Nucleotide Sequence Database (ENA) under the accession number PRJEB43415.

### Mitochondrial genome assembly and analysis

We derived mitochondrial genome sequences of *S. nebaliae* from trimmed and filtered Illumina reads. First, we ran MEGAHIT v. 1.2.9 [80] with standard settings and identified putative mitochondrial sequences by BLASTN searches (e-value cutoff ≤1e-05 [81]) against NCBI’s nt database (12-04-2020). The resulting assembly contained two mitogenomes, one of which prooved to be almost identical with the mitochondrial genome previously published for *S. nebaliae* (KP742964.1). We denoted this variant as haplotype A and kept it as is, as it was already validated in the reference study [24]. Yet, the novel mitochondrial sequence from the MEGAHIT assembly was used as a seed for subsequent reconstructions with MITObim v. 1.9.1 [82] (settings: --mismatch 0, −quick Megahit-seed) and NOVOPlasty v. 4.2 [83] (settings: genome range = 14,500–17,000, K-mer = 61, Use Quality Scores = yes, Reference sequence = SeisonMT-Haplotype). NOVOPlasty was able to reconstruct a circular sequence despite two repetitive areas in the non-coding region (NCR). Finally, all three assemblies were aligned with MAFFT v. 7 (https://mafft.cbrc.jp/alignment/server/; [84]) for manual derivation of a consensus sequence, which was verified as being a *Seison* mitogenome by BLASTN against NCBI’s nt database. We refer to this novel mitogenome as to haplotype B. Downstream gene prediction by MITOS [85] was followed by validation of protein-coding genes with NCBI ORF finder (https://www.ncbi.nlm.nih.gov/orffinder/) and subsequent BLASTP against NCBI’s non-redundant protein database. Gene boundaries were expanded with preference given to non-overlapping genes. Annotation of tRNA genes was verified with ARWEN v. 1.2 [86] and tRNAScan-SE v. 2.0 [87].

For inference of multiplicity values, we mapped the processed DNA and RNA reads (*S. nebaliae*, see RNA sequencing (RNA-seq) and de novo assembly of transcriptomes) with Bowtie 2 (http://usegalaxy.eu [88, 89]) onto both *S. nebaliae* mitogenomes. Sequence identity between the two haplotypes was derived by BioEdit v. 7.2.5 [90], after aligning with MAFFT v. 7 and revising with Gblocks v. 0.91b [91] in default settings.

A seisonid origin of both mitochondrial variants was verified by phylogenetic analyses of alignments including sequences of potential contaminants such as crustaceans or co-occuring *P. annulatus*. Limiting factor in alignment generation was the application of the same assembly pipeline as described above to trimmed *P. annulatus* reads, which resulted in a fragmented mitogenome assembly covering genes for cytochrome oxidase subunit I (*cox1*) and NADH dehydrogenase subunits 1 (*nd1*) only (see Additional file 1 for more details). In addition to the sequences of *P. annulatus* and *S. nebaliae* haplotypes A and B, the *cox1* alignment contained an additional *S. nebaliae* sequence (DQ297765.1), which had been reconstructed by another working group [37]. Due to a lack of respective sequences for *N. bipes* in GenBank, altogether three other crustacean species were taken as substitutes: *N. pseudotroncosoi* (Leptostraca; *cox1*: JX442539.1), *Pacifastacus leniusculus* (Astacoidea; *nd1*: NC_033509.1), *P.* (*Litopenaeus*) *vannamei* (Penaeoidea; cox1 and *nd1*: EF584003.1). We additionally included orthologues of the nematode *C. elegans* (*cox1 and nd1:* NC_001328.1).

Sequences were aligned with MAFFT v. 7 and pruned from uncertain alignment sections with Gblocks v. 0.91b [91]. The latter was run with default settings, except for a lowered minimum block length (5 aa). For ML tree construction with PhyML v. 3.3 [92], the best-fit substitution model (GTR + G) was determined with Smart Model Selection (SMS) v. 1.8.4 [93], applying the Akaike Information Criterion (AIC). Branch support values in ML trees give results from approximate SH-like aLRT. For BI with MrBayes v. 3.2.7a [94, 95], trees were reconstructed from posterior distributions containing every 100th generation (the first 25% were discarded as burn-in) as drawn from two runs with four chains of 1 Mio generations, each. Trees were visualized iTOL v. 5 [96].

### De novo assembly of the nuclear genome

Mitochondrial reads were excluded from reconstruction of the nuclear genome with the k-mer filtering function of BBDuk from the BBTools suite v. 38.73 (https://sourceforge.net/projects/bbmap/). Subsequent profiling with GenomeScope 1.0 [42] used Jellyfish v. 2.2.8 [97] k-mer counts (k = 21). For complementary Merqury analysis, we first determined the best k-mer size for both nuclear genome assemblies of *S. nebalia* (GA1: 99 Mb; GA2: 44 Mb) using the script best_k.sh [44]. Subsequently, we created a meryl database for the best k-mer size (k = 18) from trimmed and filtered Illumina gDNA reads. With this database, we evaluated the k-mer frequency distribution in our two nuclear genome assemblies reconstructed with MEGAHIT [80].

In a first approach (GA1), we ran MEGAHIT with default settings, apart from a stricter error control (prune-level 3). We expected GA1 to contain two nuclear genomes (A and B) of carriers of mitochondrial haplotypes A and B. We aimed to reconstruct a second assembly (GA2) that represented a single nuclear genome (B) of carriers of mitochondrial haplotype B only. To achieve this, we implemented stricter settings in error control (prune-level 3, −-no-mercy, −-disconnect-ratio 0.2, −-low-local-ratio 0.4), higher minimum k-mer size (k-min 29) and a higher minimum k-mer count and average depth (−-prune-depth 20, −-min-count 4). The implementation of a multiplicity cut-off (≥ 20 x) to GA2 implicated a filtering against less abundant k-mers. Furthermore, we scrutinized GA2 contigs for BLASTN hits with E-values ≤1e-03 and IDs ≥ 80%, and deleted manually validated hits to *N. bipes* (PRJNA67309), *Genostoma* (Platyhelminthes) and bacteria. In addition, we filtered both nuclear genome assemblies, GA1 and GA2, for a minimal contig length of 1000 bp. We used Bowtie 2 to map the filtered DNA reads to both assemblies (GA1, GA2) and derived multiplicity values for each contig. Finally, we used BUSCO v. 4.0.6 [98] to assess the level of completeness of genome assemblies GA1 and GA2. For doing so, the BUSCO pipeline was run with the metazoan database (odb10) and standard settings, except for the application of *Schistosoma mansoni* (Platyhelminthes, Trematoda) gene models. One hundred BUSCO Metazoa genes that had been classified as duplicated in GA1 were retained for further analyses, provided that the abundances of the corresponding contigs differed by 8–14-fold and orthologues were available for the other species included in downstream phylogenetic analyses. In case of the genome assemblies for *S. nebaliae* (GA2) and *P. laevis* sensu lato [23], we additionally searched for Metazoa and Eukaryota genes with the self-training function in Augustus being activated.

### Annotation of the repetitive DNA complement

For de novo generation of a custom database of *S. nebaliae* repeats, we ran RepeatModeler v. 2.0.1 [99] on the unfiltered draft genome GA2 and dnaPipeTE [100] and RepARK v. 1.3.0 [101] on all trimmed and filtered Illumina DNA reads. As recommended [102], dnaPipeTE was started with alternative read depths to find best assembly conditions. Finally, we carried out 50 runs, with a multiplicity of 0.001 (for further details on de novo repeat generation see Additional file 1). The results of all these runs were collected into a single database. Since neither RepARK nor dnaPipeTE have an integrated annotation function, repeats were annotated with TE class [103]. To avoid the masking of duplicated protein-coding genes, which were not derived from transposable elements (TEs), corresponding candidates were removed from the repeat database. For this purpose, we excluded contigs with BLASTX hits (E-value: 1e-05) to the Swiss-Prot database (release 2020_03), as long as the corresponding Swiss-Prot sequences lacked significant matches in RepBaseRepeatMaskerEdition-20,181,026 (TBLASTN; E-value: 1e-05). The repeats from all three annotations were merged into a single file. Finally, we added repeats from the RepBaseRepeatMaskerEdition-20,181,026 classified as root, Metazoa, Protostomia or Rotifera to the custom repeat database. We annotated the repeats in draft genome GA2 using RepeatMasker v. open-4.0.7, applying the more sensitive “slow search”.

### RNA sequencing (RNA-seq) and de novo assembly of transcriptomes

RNA was extracted from 120 *S. nebaliae* specimens with the TRI Reagent™ (Invitrogen™) applying the manufacturers protocol. Pelleted RNA was resolved in HPLC-grade H_2_O. Following directional library preparation, NGS used one lane of an Illumina NextSeq 500 platform (150 bp, paired-end mode, 22,878,639 pairs total). Raw reads have been deposited under ENA accession number PRJEB43415. After having verified quality of raw data with FastQC, reads were processed with Trimmomatic (see above, for versions and references; Illumina adaptor sequence trimming, LEADING:3 TRAILING:3 SLIDINGWINDOW:4:15 AVGQUAL:20 MINLEN:40). For subsequent transcriptome reconstruction, we solely used mRNA reads, which STAR v. 2.7.6a [104] had mapped to GA1 and GA2. Genome-guided transcriptome reconstruction with Trinity then ran with the aid of STAR bam files. Reconstructions additionally used the jaccard_clip flag to avoid artificial fusion of transcripts. Using these data, Trinity v. 2.11.0 [45] assembled two transcriptomes, whereby the first one should represent *S. nebaliae* strains A and B (TA1) and the second one strain B only (TA2). Running a script implemented in Trinity, we derived SuperTranscripts [105], which should combine all isoforms of individual genes into single sequences. BSUCO v. 4.0.6 [98] was used in transcript mode with the metazoan database (odb10) and *S. mansoni* (Platyhelminthes, Trematoda) gene models, to assess the completeness of both SuperTranscript sets. To achieve an approximate map of the coding sequences, TA2 transcripts were mapped to the draft genome GA2 with GMAP v. 2021-05-27 [46].

### Comparative analysis of conserved metazoan genes

Comparisons between the two *S. nebaliae* strains focused on GC content and nucleotide usage at third codon position as inferred by MEGA X [106] from 100 codon-based alignments of BUSCO metazoan genes generated with pal2nal v. 14.0 [107] and the contigs they were residing on. Pairwise comparisons of nucleotide pair frequencies and codon usage were then conducted with the MWU test implemented in SPSS v. 23 (IBM). The genes considered had to reside on contigs with abundancies differing by a factor of 8–14, which roughly corresponded to the range observed in our mitochondrial and genome assemblies. As a second criterion, the genes selected had to have orthologues in additional species included in downstream comparisons: *S. mediterranea* (Platyhelminthes; GCA_002600895.1), *B. plicatilis* (Monogononta; GCA_010279815.1), *A. vaga* (Bdelloidea; GCA_000513175.1) and *P. laevis* (Acanthocephala; GCA_012934845.1). If a gene was annotated several times as in the degenerate tetraploid *A. vaga* [20], we accepted the copy with maximum BUSCO score. Based on the BUSCO annotations for the metazoan genes kept we compared protein and gene lengths, applying the KW test in SPSS. Alpha-error rates (*p* values) from MWU and KW tests were transformed into FDRs [108].

For phylogenetic analyses, amino acid sequences were aligned separately for each of the aforementioned 100 BUSCO metazoan genes (MAFFT v. 7), followed by curation with Gblocks 0.91b (minimum block length 5). The resulting alignments were concatenated to a single dataset of 21,042 aa with six operational taxonomic units (OTUs) (Concat100-6OTUs). Applying an in-house script [19], we built three additional datasets. In one of these datasets, positions in which at least one sequence had a private character (referred to as singletons) and the outgroup representative were removed. The resulting alignment contained five OTUs and had 13,268 aa (Concat100-noSing-5OTUs). We generated further alignments which lacked the outgroup representative and one of the *S. nebaliae* strains in addition to singletons. The dataset containing strain A had 9550 aa positions (Concat100-noSing-4OTUs-A), while the one with strain B extended over 9579 aa (Concat100-noSing-4OTUs-B). Another alignment contained an ancestral sequence which we had reconstructed for the Rotifera-Acanthocephala LCA whereas the platyhelminth was deleted (Concat100-6OTUs-LCA). Reconstruction of the LCA sequence used the ML framework of CodeML implemented in PAML package v. 4.9j [109]. CodeML was run with lg.dat matrix and REVaa (189) + G + I model and the following intree: ((*P. laevis*, (*S. nebaliae* strain A, *S. nebaliae* strain B), *A. vaga*, *B. plicatilis*), *S. mediterranea*). Note that the intree was star-like for Rotifera-Acanthocephala. By doing so, reconstruction of the LCA sequence was unbiased in respect to the actual phylogenetic relationships within the clade. Based on AIC, PhyML determined LG + G + I + F as the best-fit model (SMS) for all five alignments. Tree reconstruction relied on PhyML v. 3.3 and MrBayes v. 3.2.7a once more. From two runs of four chains each was collected every 500th of a total of 100,000 generations.

We additionally compared complements of ANTP-type gene families between the monogonont *B. plicatilis* (GCA_010279815.1), *A. vaga (*GCA_000513175.1), *S. nebaliae* (present study), and *P. laevis* (GCA_012934845.1). For this purpose, we screened (TBLASTN) the (latest) genome assemblies specified in the previous sentence for aa sequences of homeodomains of various ANTP class gene homologues in species of Lophotrochozoa, Ecdysozoa and Vertebrata. Scaffolds with hits yielding incomplete homeodomains due to splice sites were analyzed with GENSCAN (Hollywood.mit.edu/GENSCAN.html) to recover complete homeodomains. First orthology assignments were made based on according BLASTX hits to NCBI’s GenBank. Results were verified by generating an alignment of the 60 aa homeodomains including sequences of homologous genes of several other species and subsequent phylogenetic analysis by FastML v. 3.1 [110] and FastTreeMP v. 2.1.10 [111] (data available upon request). Trees were visualized by FigTree v. 1.4.4 (https://github.com/rambaut/figtree/releases).

## Supplementary Information

**Additional file 1.**

**Additional file 2.**

## Data Availability

The datasets analyzed in the current study are available in the EMBL Nucleotide Sequence Database (ENA) repository under the accession number PRJEB43415 [https://www.ebi.ac.uk/ena/browser/view/ERA3506149].
