# Supplementary material for: Genomics and transcriptomics of epizoic Seisonidea (Rotifera, syn. Syndermata) reveal strain formation and gradual gene loss with growing ties to the host
Source: BMC Genomics. 2021 Aug 9;22:604. doi: 10.1186/s12864-021-07857-y (PMC8351084; doi:10.1186/s12864-021-07857-y)
Supplement: Supplementary file 1 — Additional file 1. [file 12864_2021_7857_MOESM1_ESM.pdf]

Additional File 1

More details on materials, methods and results. Assembling of *P. annulatus* mitochondrial sequences and *nd1* phylogeny; generation of custom *S. nebaliae* repeat database and repeat annotation; phylogenetic trees as inferred from 100 concatenated proteins. (PDF)

Genomics and transcriptomics of epizoic Seisonidea (Rotifera, syn. Syndermata) reveal strain formation and gradual gene loss with growing ties to the host

Mauer KM*, Schmidt H, Dittrich M, Fröbius AC, Hellmann SL, Zischler H, Hankeln T, Herlyn H*

*Corresponding authors

Content

Assembly of *Paraseison annulatus* mitochondrial genes 2

Phylogeny of Seisonidea as inferred from *cox1* and *nd1* 2

Generation of a custom *S. nebaliae de novo* repeat database 4

Annotation of the *S. nebaliae* draft genome 6

Phylogenetic trees as inferred from 100 concatenated proteins 7

Attraction to the platyhelminth and its push-away from other rotifers 8

References 9

Assembly of *Paraseison annulatus* mitochondrial genes

DNA of *Paraseison annulatus*, a close relative of *Seison nebaliae*, was subjected to whole genome amplification (WGA) prior to library construction. Subsequent gDNA-Seq and processing steps were carried out as described in the main text. Assembling with MEGAHIT v. 1.2.9 [1] resulted in several contigs with mitochondrial sequences, which were used as seed for reference-based assembly with MITObim v. 1.9.1 [2] and NOVOPlasty v. 4.2 [3]. Nevertheless, the complete mitogenome could not be assembled from the data. Probably, initial WGA let to the underrepresentation of greater parts of the mitogenome in the gDNA-Seq data. Still, major parts of *cox1* and *nd1* were contained in several contigs, each. After having aligned the corresponding sequences with MAFFT v. 7 (https://mafft.cbrc.jp/alignment/server/) [4], we manually derived a consensus sequence for both genes.

Phylogeny of Seisonidea as inferred from *cox1* and *nd1*

The aforementioned sequences were used to validate that present NGS data reflect strains within *S. nebaliae* and not contamination of our *S. nebaliae* sample with *P. annulatus* or their common crustacean host, *Nebalia bipes*. Corresponding tree reconstruction was carried out on alignments (MAFFT) including *cox1* and *nd1* sequences of *P. annulatus* in addition to their orthologues in *S. nebaliae* strains A and B (present study). The *cox1* alignment additionally contained a *S. nebaliae* sequence (DQ297765.1), which had been reconstructed by another working group [5]. We further collected sequences of the crustaceans *Nebalia pseudotroncosoi* (Leptostraca; *cox1*: JX442539.1), *Pacifastacus leniusculus* (Astacoidea; *nd1*: NC_033509.1) and *Penaeus* (*Litopenaeus*) *vannamei* (Penaeoidea; *cox1* and *nd1*: EF584003.1) and of *Caenorhabditis elegans* (NC_001328.1). All reconstructions show a monophylum including all sequences classified as *S. nebaliae* (see Table S1), when rooted by *C. elegans*. The Crustacea species used for the *nd1* and *cox1* trees were monophyletic in all phylogeny reconstructions

(Supplementary Table S1). Bayesian inference trees (MrBayes) are additionally depicted in Figure 2 (main text) and Supplementary Figure S1. For further details, see Materials and Methods in the main text.

Table S1. Trees as inferred from *cox1* and *nd1* alignments.

Dataset	Program	Newick annotation
cox1	MrBayes	(Cel,(((**SneB**,**Sne**)100,**SneA**)100,**Pan**)100,(Pva,Nps)0.548);
	PhyML	(**SneA**,(**SneB**,**Sne**)100,(**Pan**,(Cel,(Nps,Pva)100)100)100);
nd1	MrBayes	(Cel,((**SneB**,**SneA**)100,**Pan**)100,(Pva,Ple)100);
	PhyML	(((Pva,Ple)100,Cel)100,**Pan**,(**SneB**,**SneA**)100);

Node support in MrBayes and PhyML trees corresponds to posterior probabilities and results of SH-like approximate likelihood ratio test. Species abbreviations relating to Seisonidea are given in bold. *Cel*, *C. elegans*; *cox1*, cytochrome oxidase 1 gene; *nd1*, NADH dehydrogenase subunit 1 gene; *Nps*, *N. pseudotroncosoi*; *Pan*, *P. annulatus*; *Ple*, *P. leniusculus*; *Pva*, *P. vannamei*; *SneA*, *S. nebaliae* strain A; *SneB*, *S. nebaliae* strain B.

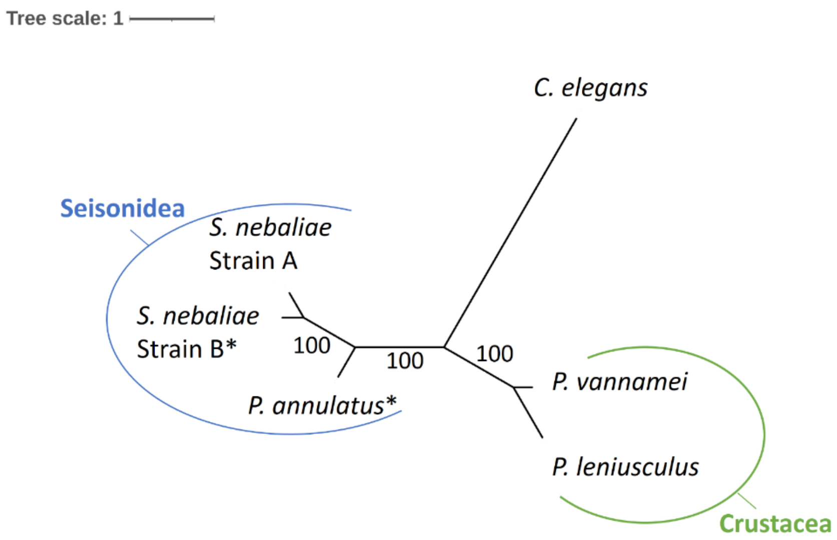

Fig. S1. Unrooted Bayesian inference tree as reconstructed from present *nd1* alignment (943 bp). Sequences of *S. nebaliae* form a monophyletic cluster being sister to the corresponding sequence in *P. annulatus*. A second cluster is comprised of the crustaceans *P. vannamei* and *P. leniusculus*. Support values correspond to posterior probabilities. Tree reconstruction was conducted with MrBayes v. 3.2.7a based on the GTR-G substitution model. Visualization used iTOL. For *COX1* tree, see main text. Asterisks (*) highlight new sequences.

Generation of a custom *S. nebaliae de novo* repeat database

For *de novo* generation of a custom database of *S. nebaliae* repeats, we ran RepeatModeler v. 2.0.1 [6], dnaPipeTE [7] and RepARK v. 1.3.0 [8]. The program dnaPipeTE [7] used a subset of trimmed and filtered forward reads for generating a Trinity assembly [9], which should result in a draft of only the repetitive genome sequences. The subset was determined by setting an estimated genome size and coverage. We used a pipeline developed by Schell *et al.* [10] to find the

best subset size to assemble the repetitive sequences as coherent as possible. While genome size was persistently set to 50 Mb, we implemented coverage values from 0.0001 to 1.3, in order to determine the best fitting one (22 increments). In doing so, we found that N50 values were highest for small coverage values (Supplementary Fig. S2).

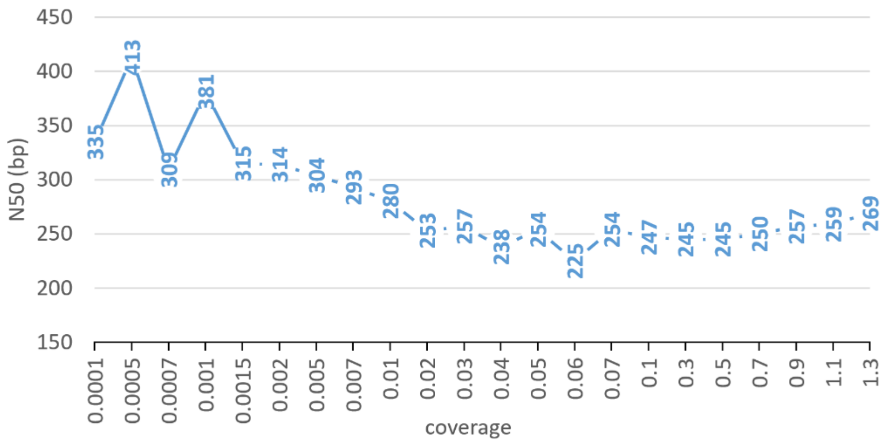

Fig. S2. N50 values of 22 dnaPipeTE runs with different coverage depths.

However, since implementation of these values might have underestimated the repetitive portion in the *S. nebaliae* genome, we decided for a larger coverage of 0.001 in combination with second largest N50 value of 381, instead of 0.005 and 413. To ensure convergence at the global maximum, we conducted 50 dnaPipeTE runs, whereby the minimum contig length was set to 200 bp. The results of all runs were merged into a single file. The program RepARK [8] was executed with default settings on trimmed and filtered forward and reverse DNA reads. In a first step, the distribution of kmers (31 bp) was inferred using Jellyfish [11] implemented in RepARK. Based on the distribution observed, RepARK defined a coverage threshold for extracting more abundant repeat-derived kmers from their counterparts representing the non-repetitive portion. The threshold determined by the program was 204, which seems plausible considering a coverage of

the homozygote portion of 132 x for GA2 (see Fig. 3 in main document). Coverage of *de novo* assembled repeat contigs (dnaPipeTE and RepARK) was determined with Bowtie2 by mapping trimmed and filtered DNA reads. In a conservative approach, contigs with an average coverage <396 were excluded from the repeat database, which is three times the coverage of the homozygote portion in the *S. nebaliae* draft genome (GA2). RepeatModeler was started with the unfiltered GA2 genome draft and the search engine RMBlast v. 2.2.27+ (http://www.repeatmasker.org/RMBlast.html), assuming a maximum genome size (-genomeSampleSizeMax) of 65 Mb. The repeats from all three annotations were eventually merged into a single file. Finally, we added repeats from the RepBaseRepeatMaskerEdition-20181026 classified as root, Metazoa, Protostomia or Rotifera to the custom repeat database. For additional references, see Materials and Methods section in the main document.

Annotation of the *S. nebaliae* draft genome

Repeats in draft genome GA2 were annotated with RepeatMasker v. open-4.0.7, applying more sensitive "slow search". Slightly fewer bases (total of 6,995,276) were masked in GA2 than reported as total span of the repetitive portion in Supplementary Table S2. This discrepancy is probably due to short stretches (less than 10 bp) between identical simple repeats, which are overstretched by annotation, but not masked in the draft genome (see http://www.repeatmasker.org/webrepeatmaskerhelp.html).

Table S2. RepeatMasker annotation of repeats in GA2.

Level 1	Count	Span [bb]	Percentage
DNA	4,915	1,042,872	2.38
LINE	7,103	1,972,450	4.49
LTR	2,366	984,955	2.24
Unclassified	11,273	1,904,047	4.34

SINE	152	13,488	0.03
Total interspersed	25,809	5,917,812	13.48
Small RNA	443	49,948	0.11
Low_complexity	3,728	180,498	0.41
Simple_repeat	21,613	959,998	2.19
Total annotated	25,784	7,108,256	16.20

Phylogenetic trees as inferred from 100 concatenated proteins

Table S3. Tabular survey of phylograms prior and after alignment editing and substitution of the outgroup by an ancestral sequence.

Dataset	Program	Newick annotation
Concat100	MrBayes	(Ava:0.262916,Pla:0.589728,(Bpl:0.313526,(Sme:0.536146,(SneA:0.045055,SneB:0.032949) 100:0.478034)100:0.049791)100:0.049455);
	PhyML	(SneA:0.04511090,SneB:0.03325317,(Sme:0.54236658,(Bpl:0.31622970,(Ava:0.26377017 ,Pla:0.59626843)100:0.04978434)100:0.04916895)100:0.48316991);
Concat100-noSing-5OTUs	MrBayes	(Bpl:0.193418,Ava:0.153258,(Pla:0.215781,(SneB:0.020456,SneA:0.017110)100:0.726982) 100:0.112410);
	PhyML	(((SneB:0.02051873,SneA:0.01622212)100:0.70448783,Pla:0.21203086)100:0.10606169, Bpl:0.18840119,Ava:0.14980406);
Concat100-	MrBayes	(Ava:0.116128,Bpl:0.151194,(Pla:0.162712,SneA:0.177262)100:0.091266);

noSing-4OTUs-A	PhyML	((Pla:0.15523360,SneA:0.17009875)100:0.08596943,Bpl:0.14240806,Ava:0.11241077);
Concat100-	MrBayes	(Ava:0.106469,Bpl:0.143125,(Pla:0.147886,SneB:0.167007)100:0.095531);
noSing-4OTUs-B	PhyML	(Ava:0.11069631,Bpl:0.14194396,(SneB:0.16799377,Pla:0.14897334)100:0.08716802);
Concat100-	MrBayes	(LCA:0.000072,Bpl:0.174284,(Ava:0.150328,(Pla:0.223263,(SneB:0.019156,SneA:0.018410)
6OTUs-LCA		100:0.738881)100:0.119894)100:0.008475);
	PhyML	(LCA:0.00000001,((Pla:0.53000942,(SneB:0.03315650,SneA:0.04430461)1.000000:0.45035597)
		1.000000:0.03159844,Ava:0.25117778)0.000000:0.00000001,Bpl:0.27972009);

Node support in MrBayes and PhyML trees corresponds to posterior probabilities and results of SH-like approximate likelihood ratio test. Ava, *A. vaga* (GCA_000513175.1); Bpl; GCA_010279815.1; *Pla*, *P. laevis* (GCA_012934845.1); *Sme*, *S. mediterranea* (GCA_002600895.1); *SneA*, *S. nebaliae* strain A (present study); *SneB*, *S. nebaliae* strain B (present study).

Attraction to the platyhelminth and its push-away from other rotifers

Seison nebaliae fell together with P. laevis upon combined removal of the playthelminth and singletons only. We see in this an indication of the other side of the coin to the attraction to the platyhelminth, namely the push-away from the other rotifers. According to this, alignment positions in which S. nebaliae has a private character state due to an exchange would tentatively support a clustering of the other OTUs. However, removal of such positions alone would not affect the

attraction between the platyhelminth and *S. nebaliae*. In turn, positions in which *S. nebaliae* and the platyhelminth have identical character states (by independent exchanges) cause their clustering. But removal of the platyhelminth alone would not touch the attraction between the other OTUs.

References

1. Li D, Liu CM, Luo R, Sadakane K, Lam TW. MEGAHIT: an ultra-fast single-node solution for large and complex metagenomics assembly via succinct de Bruijn graph. Bioinformatics. 2015;31:1674–1676.

2. Hahn C, Bachmann L, Chevreux B. Reconstructing mitochondrial genomes directly from genomic next-generation sequencing reads – a baiting and iterative mapping approach. Nucleic Acids Res. 2013;41:e129.

3. Dierckxsens N, Mardulyn P, Smits G. NOVOPlasty: *de novo* assembly of organelle genomes from whole genome data. Nucleic Acids Res. 2016;45:e18.

4. Katoh K, Standley DM. MAFFT multiple sequence alignment software version 7: improvements in performance and usability. Mol Biol Evol. 2013;30:772–780.

5. Sørensen M V., Giribet G. A modern approach to rotiferan phylogeny: combining morphological and molecular data. Mol Phylogenet Evol. 2006;40:585–608.

6. Smit A, Hubley R. RepeatModeler Open-1.0. Available at: http://www.repeatmasker.org

7. Goubert C, Modolo L, Vieira C, Moro CV, Mavingui P, Boulesteix M. *De novo* assembly and annotation of the Asian tiger mosquito (*Aedes albopictus*) repeatome with dnaPipeTE from raw genomic reads and comparative analysis with the yellow fever mosquito (*Aedes aegypti*). Genome Biol Evol. 2015;7:1192–1205.

8. Koch P, Platzer M, Downie BR. RepARK – *de novo* creation of repeat libraries from whole-genome NGS reads. Nucleic Acids Res. 2014;42:e80.

9. Grabherr MG, Haas BJ, Yassour M, Levin JZ, Thompson DA, Amit I, et al. Full-length transcriptome assembly from RNA-Seq data without a reference genome. Nat Biotechnol. 2011;29:644–652.

10. Schell T, Feldmeyer B, Schmidt H, Greshake B, Tills O, Truebano M, et al. An annotated draft genome for *Radix auricularia* (Gastropoda, Mollusca). Genome Biol Evol. 2017;9:585–592.

11. Marçais G, Kingsford C. A fast, lock-free approach for efficient parallel counting of occurrences of k-mers. Bioinformatics. 2011;27:764–770.
